# Integrated promoter-capture Hi-C and Hi-C analysis reveals fine-tuned regulation of the 3D chromatin architecture in colorectal cancer

**DOI:** 10.3389/fgene.2025.1553469

**Published:** 2025-03-28

**Authors:** Ajay Kumar Saw, Ayush Madhok, Anupam Bhattacharya, Soumyadeep Nandi, Sanjeev Galande

**Affiliations:** ^1^ Laboratory of Chromatin Biology and Epigenetics, Department of Biology, Indian Institute of Science Education and Research, Pune, India; ^2^ Division of Life Sciences, Institute of Advanced Study in Science and Technology, Vigyan Path, Paschim Boragaon, Garchuk, Guwahati, Assam, India; ^3^ Department of Molecular Biology and Biotechnology, Cotton University, Panbazar, Guwahati, Assam, India; ^4^ Data Sciences and Computational Biology Centre, Amity Institute of Integrative Sciences and Health, Amity University Haryana, Gurugram, Manesar, Haryana, India; ^5^ Center of Excellence in Epigenetics, Department of Life Sciences, Shiv Nadar University, Gautam Buddha Nagar, Uttar Pradesh, India

**Keywords:** colorectal cancer, topologically associated domains, genome organization, epigenome, gene expression, biomarker

## Abstract

**Introduction:**

Hi-C is a widely used technique for mapping chromosomal interactions within a 3D genomic framework, however, its resolution is often constrained by sequencing depth, making it challenging to detect fine-scale interactions. To overcome this limitation, Promoter-Capture Hi-C (PCHi-C), as it selectively enriches for promoter-associated interactions, was employed. This study integrates PCHi-C and Hi-C datasets from colorectal cancer (CRC) models investigate chromosomal interaction dynamics across various regulatory levels, from cis-regulatory elements to topologically associated domains (TADs). The primary goal is to examine how genomic structural alterations shape the epigenomic landscape in CRC and to assess their potential role in colorectal cancer susceptibility.

**Methods:**

PCHi-C and Hi-C datasets from multiple colorectal cancer (CRC) studies were integrated to enhance the resolution of chromatin interaction mapping. The analysis focused on identifying fine-scale interactions within topologically associated domains (TADs) while incorporating histone modification landscapes (H3K27ac, H3K4me3) and transcriptomic signatures from CRC cell lines and the TCGA database. For experimental validation, ChIP-quantitative PCR was performed at the promoters of target genes using the highly malignant colorectal cell line HT29 and compared it to an embryonic cell line NT2D1.

**Results:**

Our integrated analysis revealed significant genomic structural instability in CRC cells, closely associated with tumor-suppressive transcriptional programs. We identified nine dysregulated genes, including long non-coding RNAs (MALAT1, NEAT1, FTX, and PVT1), small nucleolar RNAs (SNORA26 and SNORA71A), and protein-coding genes (TMPRSS11D, TSPEAR, and DSG4), all of which exhibited a substantial increase in expression in CRC cell lines compared to human embryonic stem cells (hESCs). Additionally, we observed enriched activation-associated histone modifications (H3K27ac and H3K4me3) at the potential enhancer regions of these genes, indicating possible transcriptional activation. ChIP-quantitative PCRs conducted using in the highly malignant CRC cell line HT29, compared to the embryonic cell line NT2D1, further validated these findings, reinforcing the link between altered chromosomal interactions and gene dysregulation in CRC.

**Discussion:**

This study sheds light on the dynamic 3D genome organization in CRC, highlighting critical structural changes associated with disease-associated loci. The identification of nine dysregulated genes points to potential biomarkers for colorectal cancer, with implications for diagnostic and therapeutic strategies. The combination of Hi-C and PCHi-C offers a refined approach for detecting chromosomal interactions at a higher resolution, laying the foundation for future studies on cancer-associated chromatin architecture.

## 1 Introduction

Until recently, studies on the impact of three-dimensional changes on gene expression regulation were restricted due to the limited tools available to investigate the three-dimensional genome structure. Cutting-edge techniques for unraveling chromatin organization have enabled explorations into the gene expression mechanisms in a new dimension. Techniques such as fluorescence *in situ* hybridization (FISH) (Bienko et al., 2013; Beliveau et al., 2012) and chromosome conformation capture (3C, 4C, 5C, Hi-C, and promoter-capture Hi-C) have been developed for studying the mechanisms of gene expression. Using these methods, several studies (Dekker et al., 2002; Lieberman-Aiden et al., 2009; Zhao et al., 2006; Rao et al., 2014; Freire-Pritchett et al., 2021) have revealed hierarchical layers of spatial organization of genomes, further unveiling that the 3D genome architecture is highly dynamic throughout the course of an animal’s development. Chromosome conformation capture methods, such as Hi-C and promoter-capture Hi-C (PCHi-C), provide averaged 3D genomic interaction information derived from their application to millions of cells. Hi-C maps have been generated across a variety of human cell categories, encompassing embryonic stem cells and early embryonic lineages (Dixon et al., 2012; Dixon et al., 2015; Pelham-Webb et al., 2020; Djeghloul et al., 2020; Du et al., 2020; Collombet et al., 2020), immune cells (Rao et al., 2014), fibroblasts (Jin et al., 2013), and other primary tissue types (Schmitt et al., 2016). Hi-C^4^ enables whole genome-wide mapping of long-range chromatin interactions and, therefore, represents a powerful approach for predicting the distal gene targets of disease-associated loci. Hi-C-derived methodologies are advancing our understanding of TAD-level organization, A/B compartments, and loop formations (Klein et al., 2021; Fukuda et al., 2021; Spracklin et al., 2021; Hildebrand and Dekker, 2020; van Schaik et al., 2020; Su et al., 2020). However, effectively identifying intra-TAD interactions, such as regulatory loops, from Hi-C data remains a challenging task due to the complexity of the Hi-C libraries and the considerable expense associated with deep sequencing to obtain statistically significant interactions. On the other hand, targeted chromatin-capture techniques such as PCHi-C provide cis-regulatory insights for a specific subset of clinically relevant genomic regions at a substantially lower cost with fine-scale genomic interactions. High-resolution maps of clinically relevant loci enable more accurate predictions of the impacts of structural changes and alterations that may lead to human diseases or developmental abnormalities (Lupiáñez et al., 2016). PCHi-C significantly enhances the capability to identify interactions that encompass promoter sequences. PCHi-C analysis across distinct cell types recognized numerous enhancer–promoter interactions and unveiled significant differences in promoter architecture between cell types and during differentiation (Schoenfelder et al., 2015; Mifsud et al., 2015; Javierre et al., 2016; Freire-Pritchett et al., 2017; Rubin et al., 2017; Siersbæk et al., 2017). These investigations demonstrated that the organization of the genome reflects cellular identity, underscoring the importance of disease-relevant cell types toward deciphering the gene regulatory mechanisms associated with disease loci. To support this idea, several recent studies have utilized high-resolution promoter interaction maps to identify tissue-specific target genes associated with GWAS findings. An examination of promoter-capture Hi-C data across 17 primary human blood cell types captured 2,604 potentially significant genes related to immune and blood-related disorders, including a considerable number of genes with roles yet to be annotated in those diseases (Javierre et al., 2016). Montefiori et al. generated high-resolution PCHi-C mapping in human-induced pluripotent stem cells (iPSCs), and iPSC-derived cardiomyocytes (CMs) unveiled 1,999 single-nucleotide polymorphisms (SNPs) associated with cardiovascular diseases, which were linked to 347 target genes. This highlights the significance of incorporating long-range chromatin interactions into the interpretation of functional targets associated with disease loci (Montefiori et al., 2018). Philip et al. performed a GWAS meta-analysis to explore the gene regulatory mechanisms underpinning all GWAS risk loci. They achieved this by analyzing PCHi-C datasets and capturing chromatin interactions between predisposition loci and target genes. They also scrutinized gene expression data and integrated these findings with chromatin immunoprecipitation-sequencing (ChIP-seq) data for 31 newly identified loci, in addition to the previously known loci, to investigate the heritable risk of colorectal cancer susceptibility (Law et al., 2019).

**FIGURE 1:**
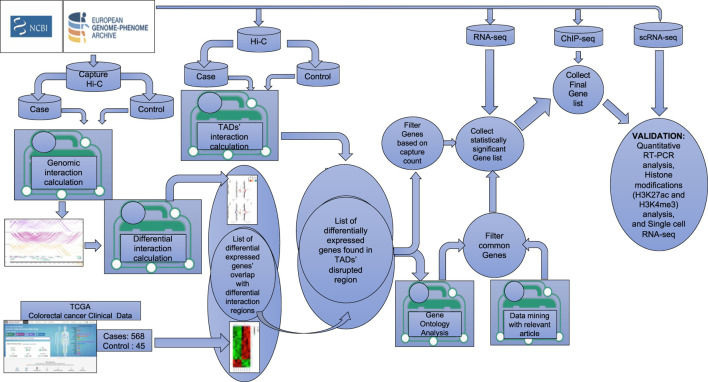
Schematic diagram of the workflow for the identification of target genes. Graphical representation of the steps used in the analysis combining multiple large colorectal cancer datasets (PCHi-C, Hi-C, ChIP-seq, RNA-seq, and single-cell RNA-seq) to identify target genes potentially playing a key role in CRC progression.

Colorectal adenocarcinoma, the fourth most common epithelial tumor, continues to be the predominant cause of mortality worldwide (Bray et al., 2018; Alzahrani et al., 2021). In order to acquire detailed genome architectural insights into the progression of colorectal tumors, it is essential to establish a comprehensive gene regulatory map of human colon cells. This will facilitate an understanding of how TAD disruptions influence gene regulation. In this study, we present an integrative approach to comprehensively detect structural changes in cancer genomes by combining Hi-C, PCHi-C, RNA-seq, ChIP-seq, and single-cell RNA-seq (scRNA-seq) analyses for the identification of the multiple target genes that may potentially serve as biomarkers for CRC susceptibility (the strategy is depicted in Figure 1). We further demonstrate that the expression of these genes, along with the associated activatory chromatin modifications, such as H3K27ac and H3K4me3, at the target regions is upregulated in CRC. In summary, this study illustrates the dynamic interplay between global and local chromatin architecture. Furthermore, by integrating chromatin architecture with gene expression and chromatin modification profiles, we identified novel regions of colorectal cancer susceptibility.

## 2 Materials and methods

### 2.1 Promoter-capture Hi-C data processing and analysis

Raw PCHi-C sequencing reads were aligned to the hg38 human reference genome and filtered using HiCUP (S et al., 2015) and Bowtie2 (Langmead and Salzberg, 2012). To identify regions that interacted with promoters, referred to as promoter-interacting regions (PIRs), we utilized the CHiCAGO tools (Cairns et al., 2016). Promoter-interacting regions were identified by comparing the number of promoter-ligated regions within each genomic bin to the expected reads, according to the generated model. Genomic bins with CHiCAGO scores ≥5 were considered to be PIRs. BAM files were converted into a CHiCAGO-compatible format (.chinput) using bam2chicago script. Chinput files are tab-delimited text files that contain essential information of all pairs of promoter–“other end” regions for which at least one ligation region was detected. Each line in the .chinput file provides details about one promoter–“other end” pair, including their respective genomic coordinates.

### 2.2 Differential chromosomal interaction analysis using PCHi-C data

Detecting differential signals in sequencing data is a fundamental and frequently performed task in genomic analyses. We used Chicdiff (Cairns et al., 2019) for the differential analysis of significant interactions identified by CHiCAGO between different cell types. This tool integrates moderated differential testing for count data using negative binomial generalized linear models implemented in DESeq2 (Love et al., 2014), with signal normalization informed by CHiCAGO and non-uniform multiple testing correction.

### 2.3 Hi-C data processing and analysis

Reads were aligned to the human reference genome hg38 using Bowtie2 (Langmead and Salzberg, 2012), and then SAMtools (Li et al., 2009) was used to convert the reads to the BAM format. We used the HiCExplorer package (Wolff et al., 2020; Ramírez et al., 2018) for Hi-C data processing. The “hicBuildMatrix” command was used to construct the matrix of read counts over the genomic bins, taking into account sites around the given restriction site (AAGCTT). The matrix was corrected using the “hicCorrectMatrix” command to eliminate biases related to GC content, open chromatin, and, most importantly, normalize the number of restriction sites per bin. Topologically associated domains (TADs) were identified using the “hicFindTADs” command with specific parameters, including “–minDepth 60,000,” “–maxDepth 120,000,” and “–step 20,000.” The visualization of the interaction matrix and other signal tracks was performed using the “hicplotsTADs” command. The positions of TAD boundaries were used to analyze the localization of identified genomic interactions relative to these TAD boundaries.

### 2.4 ChIP-seq data processing and analysis

ChIP-seq reads were trimmed using the Trim Galore tool for adapter removal. Filtered reads were mapped to the human reference genome hg38 using Bowtie2 (Langmead and Salzberg, 2012), and then SAMtools (Li et al., 2009) was used to convert the reads to the BAM format. macs2_callpeak was used for peak calling with –tsize = 26, –gsize = 2.7e9, and –extsize 200, with the remaining settings left as default. The MACS2 tool is designed to account for the impact of genome complexity when assessing the significance of enriched ChIP regions. It enhances the spatial resolution of binding sites by incorporating information from both the position and orientation of sequencing tags.

### 2.5 RNA-seq data processing and analysis

#### 2.5.1 RNA-seq pipeline 1

The gene expression abundance from paired-end RNA-seq datasets was downloaded from The Cancer Genome Atlas (TCGA) data portal. For the retrieval of colon cancer datasets, primary sites “colon” and “rectum,” project IDs “TCGA-COAD” and “TCGA-READ,” and sample types “primary tumor,” “tumor,” and “metastatic” were selected. For the control datasets, “blood-derived normal” and “solid tissue normal” parameters were selected during the data retrieval process. The sample manifest file was prepared, and GDC client (Morgan and Davis, 2017) was used for downloading the datasets.

#### 2.5.2 RNA-seq pipeline 2

Quality control of the dataset and mapping of processed reads were carried out as follows:

Raw files were downloaded and converted using the SRA toolkit. After that, the QC program was used to filter low-quality reads and remove any sequencing errors. The processed reads were aligned using the RNA-seq aligner STAR (v2.5)^42^ with the Ensembl human reference annotation (GRCh38).

#### 2.5.3 Quantifying transcript abundances

RSEM (Li and Dewey, 2011) was employed to quantify transcript abundance from the paired-end RNA-seq datasets. The human reference genome was built using the rsem-prepare-reference script. We used STAR to perform transcriptome-based mapping, and gene expression levels were calculated from STAR-generated BAM files by rsem-calculate-expression scripts.

#### 2.5.4 Identification of differentially expressed genes

The edgeR package (v3.30.0)^44^, which relies upon count-based expression data for determining differential expression in R, was used for differential gene expression analysis. Transcripts with zero expression values were removed. Normalization of the counts was performed using the calcNormFactors function of edgeR, which normalizes for differences in RNA composition by finding a set of scaling factors for the library sizes that minimize the log-fold changes between samples for most genes. The TMM method of normalization was used for the datasets.

#### 2.5.5 RNA-seq pipeline 3

The preprocessed RNA transcripts were mapped to *Homo sapiens* (Gencode v29) transcriptomes using Salmon (Patro et al., 2017). The resulting quant files were imported into R (v4.0.3) for exploratory data analysis. R package tximeta (Love et al., 2020) was used to import and summarize transcript-level data to the gene level. DESeq2 (Love et al., 2014) was used for differential expression analysis with the default setting. Further details of the used pipeline are available at the following link: (https://bioconductor.org/packages/release/workflows/vignettes/rnaseqGene/inst/doc/rnaseqGene.html).

### 2.6 GO analysis, network analysis, and mutational analysis details

The STRING database was used for the functional annotation of genes. During the analysis, GO biological process libraries 2021 and molecular function libraries 2021 were selected. The statistically significant results (p-value ≤ 0.05) were further considered for downstream analysis as a reference organism for the annotation of genes. The enriched gene clusters were visualized in the STRING network. We curated the function of genes based on functional enrichment analysis associated with key functions such as chromatin remodeling, chromatin organization, and cellular organization, among others. For mutational analysis, the Colon Cancer Atlas and the COSMIC database were used as reference databases, containing data from 13,711 CRC tissues and more than 165 CRC cell lines.

### 2.7 Single-cell RNA-seq data processing and analysis

We used the Seurat package (Stuart et al., 2019) (v4.0.6) for this integrative multimodal analysis. Genes detected in fewer than 100 cells, cells exhibiting expression of less than 200 detected genes, and cells expressing >15% mitochondrial genes were removed for downstream analysis. We adopted the general protocol described by Stuart et al. (2019) for grouping single cells into different cell subsets. We employed the following steps: data normalization, identification of highly variable features, scaling the data, clustering the cells, reordering the clusters based on their similarity, running non-linear dimensional reduction (tSNE), and labeling the cell types. Principal component analysis (PCA) was carried out on the scaled data of highly variable genes. The first 30 principal components (PCs) were used to cluster the cells and perform a subtype analysis by non-linear dimensionality reduction (t-SNE) (Van Der Maaten, 2014). Additionally, we utilized the SingleR package (v1.8.1) for labeling cell types (Aran et al., 2019).

### 2.8 Cell lines and culture

NT2D1 embryonic cells (gifted by Dr. Peter Andrews) were cultured in DMEM (Invitrogen), with 20% FBS (Invitrogen), 1X antibiotics (Antibiotic-Antimycotic, Invitrogen) and NEAA supplements (Lonza) at 37o C with 5% CO_2_. The cells were split after every 3 days and maintained at 70% confluency to avoid any differentiation. HT29 colorectal carcinoma cell line (ATCC, HTB-38) was cultured in DMEM, with 10% FBS and antibiotics. Cells were split after every 1.5 days. Similar cell culture regime was followed with a change to RPMI-1640 media (Thermo Fisher Scientific) for other colon cancer lines used including DLD1 (ATCC, CCL-221) COLO-205 (ATCC, CCL-222) and HCT-115 ( ATCC, CCL-225). FHC (fetal human colon) and hESC (human embryonic stem cells) were used as control cell lines, while HT29 and LoVo served as treated cell lines. We compared FHC and hESC with the cancer cell lines (HT29 and LoVo) to evaluate their similarity in terms of changes in Topologically Associated Domains (TADs). The primary objective was to assess whether hESC could function as a control for further downstream analyses. Two or 10 million cells were harvested for RNA extraction and chromatin immunoprecipitation assays, respectively.

### 2.9 RNA extraction and quantitative RT-PCR

The cells harvested (2 × 10^6^) were washed twice with cold 1X PBS, and the pellets were re-suspended in TRIzol (Invitrogen), followed by the isolation of total RNA. Following DNase I (Promega) digestion, RNA (260/280 ratio ∼2) was subjected to cDNA synthesis using a high-capacity cDNA synthesis kit (Applied Biosystems), as per the manufacturer’s protocol. Target oligos were designed using the UCSC Genome Browser and Primer3 software. The expression level of h18s RNA was used as the internal control for the normalization of target transcripts. Quantitative RT-PCR analyses were performed using TB Green II qPCR Master Mix (TaKaRa) with the following PCR conditions: step 1, 95°C for 5 min; step 2, 95°C for 45 s, 60°C for 45 s, and 72°C for 1 min, repeated for 40 cycles, using the ViiA 7 Real-Time PCR system (Applied Biosystems). The change in gene expression was calculated using the formula ΔCt = Ct target − Ct control. Normalized transcript expression was calculated using the equation; 2−(ΔCt), where ΔCt = Ct target − Ct control. The oligonucleotide primer sequences used for qRT-PCR analyses are listed in Supplementary Table S1. Statistical analysis was performed using one-way ANOVA (GraphPad v9.1) on three biological replicates.

### 2.10 Chromatin immunoprecipitation q-PCR

Cells obtained were cross-linked using 1.25% formaldehyde (Sigma), followed by quenching with 150 mM glycine. Cross-linked cells were washed twice with PBS and subjected to chromatin isolation and shearing, as described by Patta et al. (2020), with a few modifications. In brief, nuclei were isolated using the hypotonic buffer (25 mM Tris–HCl at pH 7.9, 1.5 mM MgCl_2_, 10 mM KCl, 0.1% NP-40, 1 mM DTT, 0.5 mM PMSF, and 1× protease inhibitor cocktail) (Roche). Pelleted nuclei were lysed using the sonication buffer (50 mM Tris–HCl at pH 7.9, 140 mM NaCl, 1 mM EDTA, 1% Triton X-100, 0.1% sodium deoxycholate, 0.5% SDS, 0.5 mM PMSF, and 1× protease inhibitor cocktail) (Roche), and chromatin was subjected to sonication using the Covaris M220 sonicator with the following parameters: peak power 75, duty factor 20, burst 300, and duration 10 min. We obtained the chromatin fragment size of 200–400 bp. After pre-clearing, chromatin was subjected to immunoprecipitation using anti-H3k27ac (Abcam) and anti-H3K4me3 (Abcam) antibodies overnight at 4°C. Similarly, normal IgG was used as a control. Immunoprecipitated complexes were pulled down by adding protein A/G beads (Thermo Fisher Scientific), and the cocktail was incubated at 4°C for 4 h. The immunoprecipitated bead-bound chromatin was washed thoroughly using low-salt, high-salt, and lithium chloride buffers and subjected to elution using the elution buffer (1% SDS, 0.1 M NaHCO_3_). The eluted chromatin was de-crosslinked, and protein and RNA contamination were removed by treating with proteinase K (Sigma-Aldrich) and RNase A (Sigma-Aldrich), respectively. Furthermore, the immunoprecipitated chromatin was purified and subjected to the quantitative PCR analysis using the formula ΔCt = Ct _Target_ − Ct _Input_. Fold differences in enrichment were calculated using equation 2^−(ΔCt)^, where ΔCt = Ct _Target_ − Ct _Input_, for both IgG- and TCF1-immunoprecipitated DNA. The primer sequences used for ChIP-PCR analysis are listed in Supplementary Table S2. Statistical analysis was performed using one-way ANOVA (GraphPad v9.1) with three biological replicates.

## 3 Results

### 3.1 hESC and FHC cell lines share high percentage of common genes with HT29 and LoVo CRC cell lines in TADs disruption comparison

**FIGURE 2:**
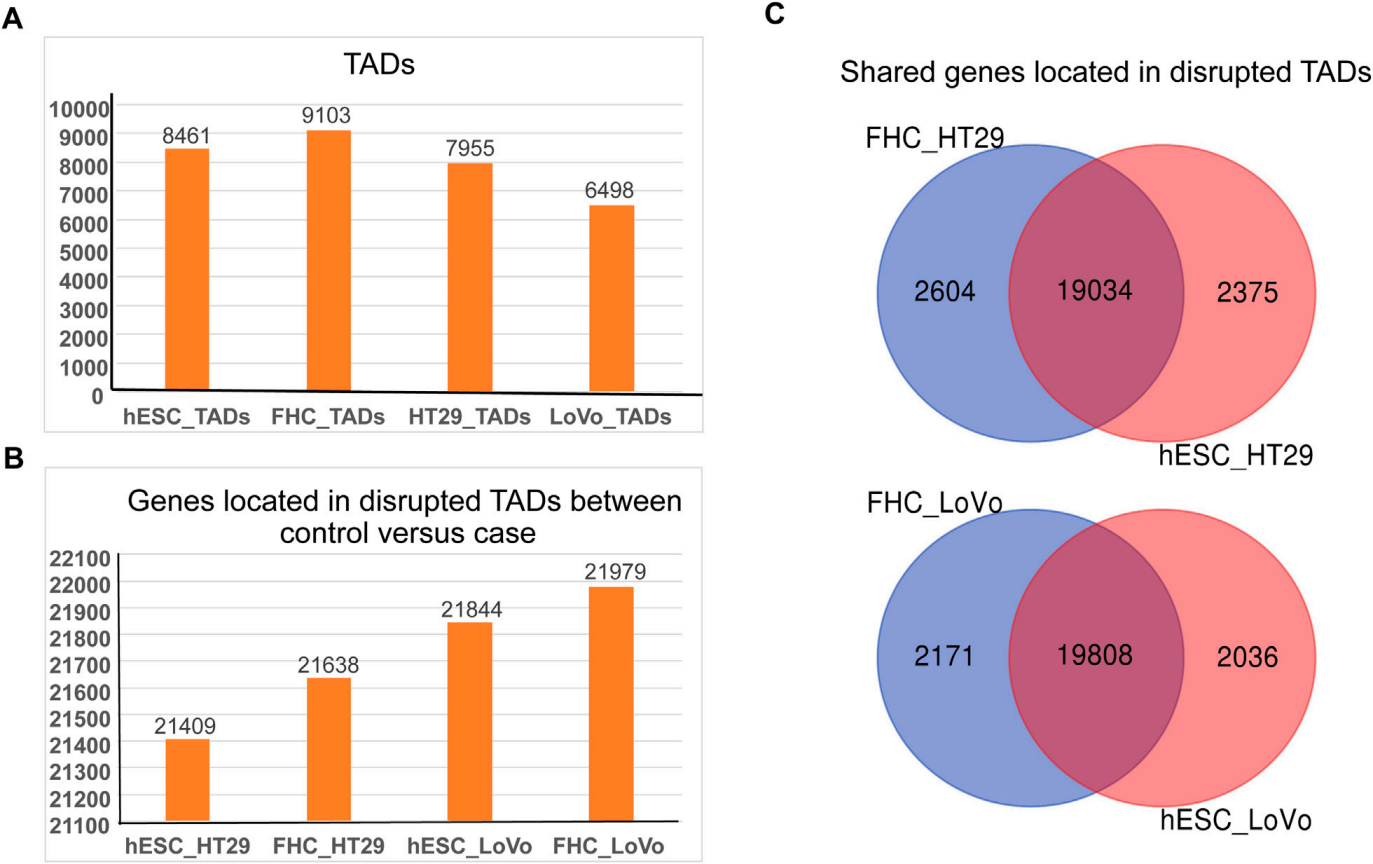
Comparison of the distribution of TADs and genes within TADs between case cell lines (HT29 and LoVo) and control cell lines (hESC and FHC). Here, TAD refers to intra-chromosomal interaction between two different genomic fragments. **(A)** Number of TAD boundaries against hESC, FHC, HT29, and LoVo cell lines generated by HiCExplorer. **(B)** Number of genes located within disrupted TADs in control (hESC and FHC) *versus* case (HT29 and LoVo) cell lines. **(C)** Venn diagram depicting genes within disrupted TADs between control and case groups.

We performed comparative study of FHC (fetal human colon) and hESC (human embryonic stem cell) lines to understand the chromatin architectural differences between FHC and hESC versus the CRC cell lines (HT29 and LoVo). The basis for selecting conserved Topologically Associated Domains (TADs) was ensuring that the genes within the overlapping TADs matched those in their respective TADs. Consequently, overlapping TADs that did not meet this criterion were regarded as disrupted TADs. Towards this, we filtered affected genes which have been found in TADs disrupted regions in FHC & hESC versus CRC (HT29 & LoVo) cell lines. HiCExplorer39 has been used to search for topological associated domains (TADs) (Figure 2A). Figure 2B depicts the number of genes that have been found in TADs disruption regions in FHC & hESC versus CRC (HT29 & LoVo) cell lines. Further comparative analyses revealed 79% common genes within the TADs disruption region in hESC versus HT29 and FHC versus HT29 comparison and 83% common genes lies in the TADs disruption region in hESC versus LoVo and FHC versus LoVo cells. This indicates that TADs generated using FHC and hESC datasets have maximum gene similarity with the CRC cell in the TADs disruption region. However, due to the limited depth of sequencing of Hi-C datasets, we were unable to capture fine scale interactions within TADs and therefore it is difficult to integrate fine-scale interaction with histone modification marks and transcriptional signature. In such a scenario, promoter-capture Hi-C (PCHi-C) data is often used for mapping fine scale interactions. Currently, PCHi-C data is available only for hESC, HT29 and LoVo, not FHC cells in publicly available databases. We performed a comparative study of hESC and FHC against CRC cell lines (Figure 2C) to capture and compare the subset of common genes in the TADs disruption region. We obtained 79% and 83% common genes in TADs disruption loci respectively (Figure 2C), showing good similarity between hESC and FHC cell lines while comparing with CRC cell lines in the context of TADs disruption. Since the PCHi-C data is not available for FHC, we therefore chose to use hESC instead FHC for further comparative study. Thus, we used hESC as a control and HT29 and LoVo as a case for capturing the fine-scale interactions and association with epigenomic marks for novel biomarker identification in CRC progression.

### 3.2 Enhancer–promoter interactions are enriched in HT29 and LoVo cell lines compared to hESC, which corresponds to changes in gene expression dynamics

**FIGURE 3:**
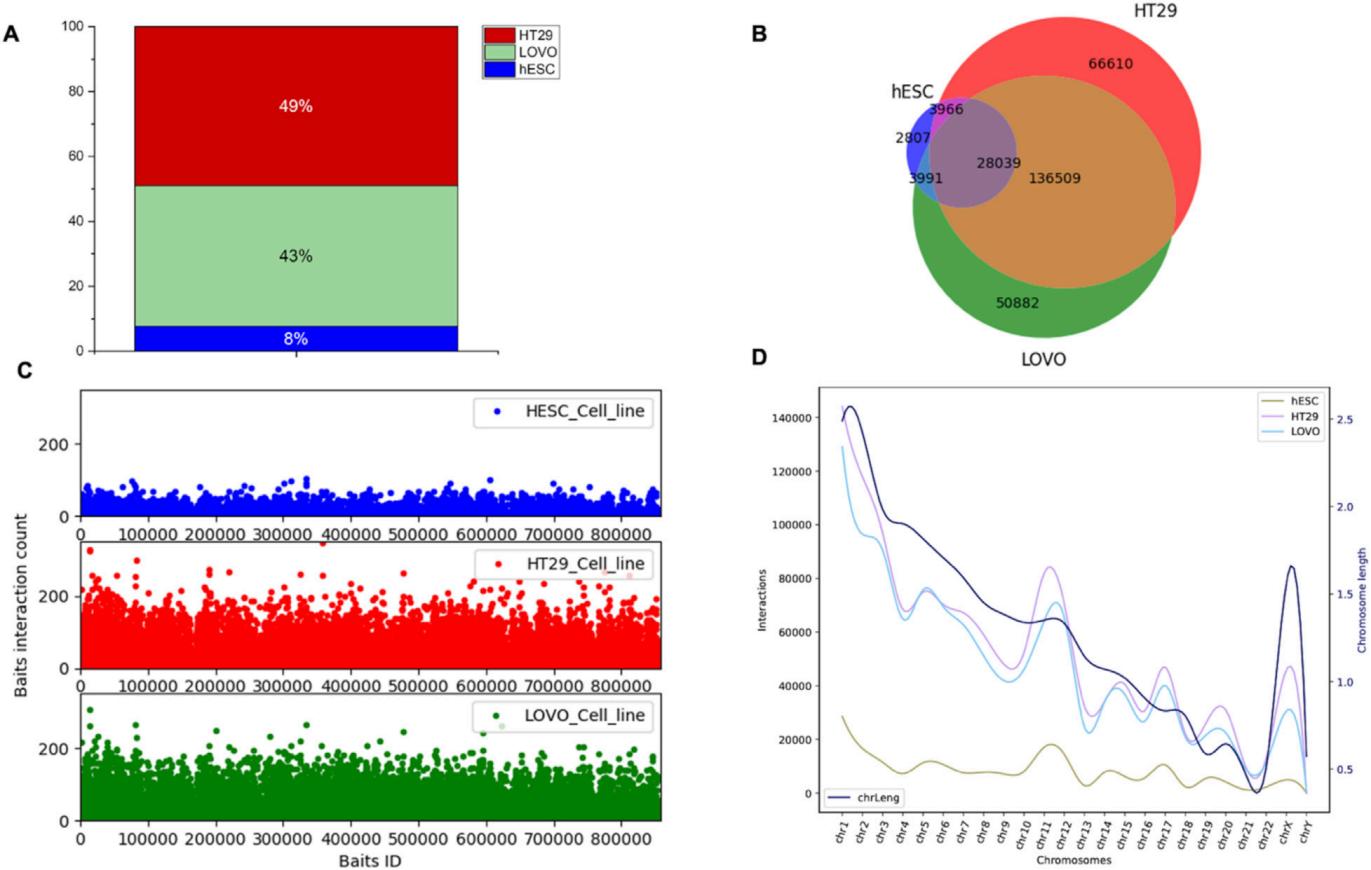
Distribution of promoter-capture Hi-C interactions in hESC, HT29, and LoVo cell lines. Here, bait fragment refers to the promoter-capture region or region of interest and other-end fragment refers to those fragments that interact with the bait fragment. **(A)** Proportion of bait interactions with other ends generated by CHiCAGO (Cairns et al., 2016) using PCHiC data of HESC, HT29, and LoVo cell lines. **(B)** Venn diagram displaying the number of bait fragments among hESC, HT29, and LoVo. **(C)** Interaction counts of bait fragments with other-end fragments of hESC, HT29, and LoVo cell lines. **(D)** Chromosome-wise interaction counts of bait fragments with other-end fragments of hESC, HT29, and LoVo cell lines. The black curve depicts chromosome lengths as per the reference genome GRCh38.

**FIGURE 4:**
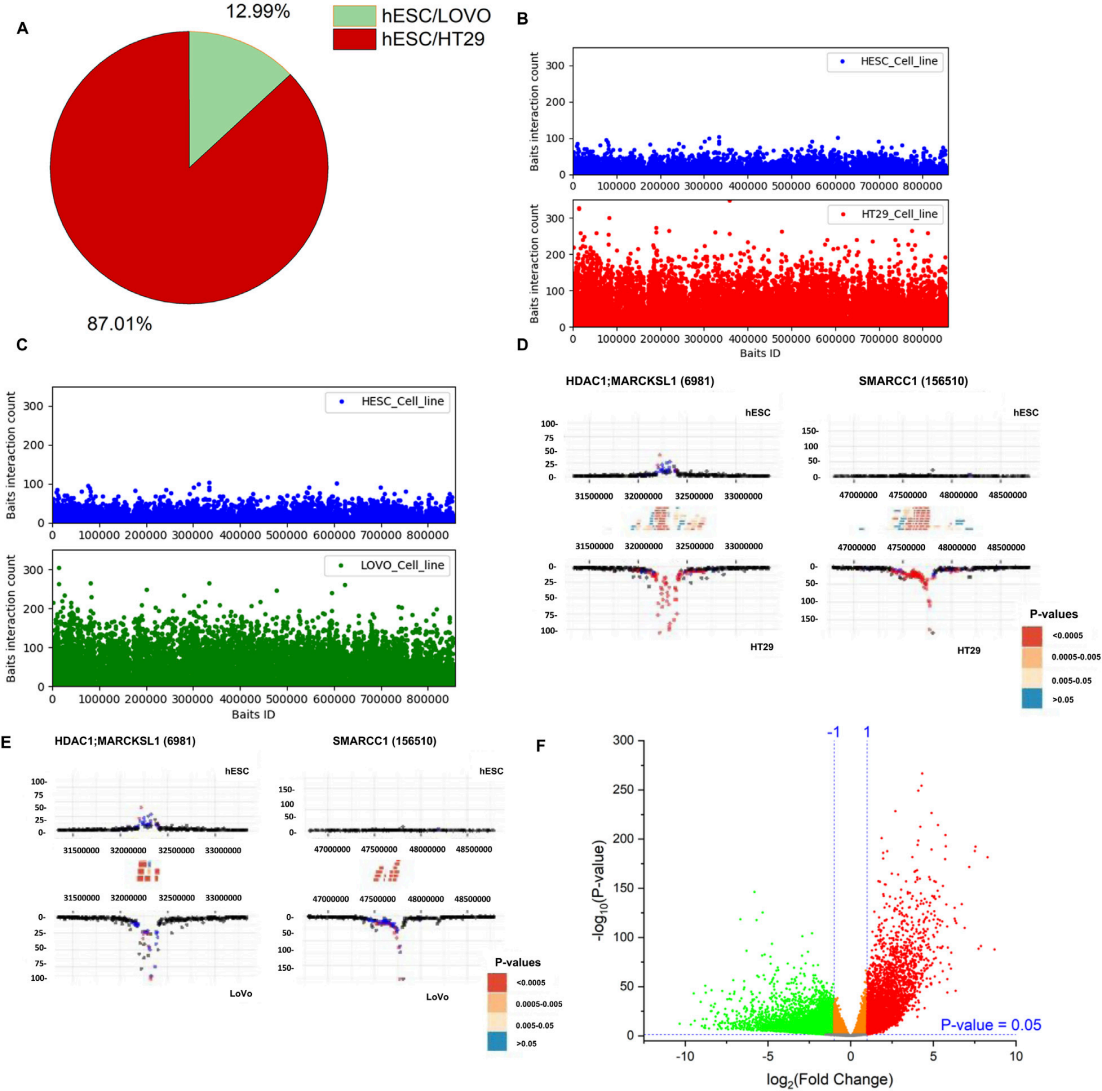
Distribution of differential promoter-capture Hi-C interactions between cancer cell lines (HT29 and LoVo) *versus* normal cell line (hESC). **(A)** Pie-chart depicting proportions of differential bait interactions between cancer *versus* normal cell lines. **(B, C)** Differential bait interaction counts of cancer *versus* normal cell lines with respect to bait fragments. **(D, E)** Differential interaction detected by Chicdiff (Cairns et al., 2019) around HDAC1, MARCKSL1, and SMARCC1 genes are plotted. Significant interactions were detected for each condition separately by CHiCAGO (Cairns et al., 2016) and color-coded (blue: 3 < score ≤ 5; red: score >5). Significant differentially interacting regions detected by Chicdiff (Cairns et al., 2019) were depicted as red blocks against HT29 *versus* hESC and LoVo *versus* hESC cell lines. **(F)** Volcano plot of differentially expressed genes generated from the colorectal cancer dataset from TCGA database. A total of 57,864 transcripts were observed. A total of 27,676 statistically significant transcripts were identified (false discovery rate ≤ 0.05), out of which 17,146 transcripts were expressed in cancer cells, while 10,530 transcripts were expressed in normal cells.

We used PCHi-C data to examine genomic interaction in CRC. The captured regions (genomic region of interest or diseases-associated loci) are referred to as “baits” throughout the manuscript. In this study, we selected two colon cancer cell lines (HT29 and LoVo) and compared them with human embryonic stem cells (hESCs). We used CHiCAGO (Cairns et al., 2016) to identify significant interactions. We investigated the interactions that were separated by a distance of at least 10 Kb. We identified bait interactions with other genomic loci, and the results are shown in Figure 3. In total, we identified 1,283,056 (49%) and 1,135,609 (43%) bait interactions in HT29 and LoVo cell lines, respectively, and 201,356 (8%) interactions in the hESC cell line (Figure 3A). Most interactions were found in cancer cell lines (HT29 and LoVo) compared to those in hESCs. A significant proportion of bait interactions was shared between the cancer cell lines compared to those in hESCs (Figure 3B). We also calculated the counts of interactions with respect to each bait fragment and chromosome-wise across all the cell lines (Figures 3C,D). We observed similar interaction patterns in HT29 and LoVo cell lines compared to those in hESCs (Figure 3D). We used Chicdiff (Cairns et al., 2019) for differential bait interaction between cancer and normal cell lines. We plotted the distribution of differential bait interactions (Figures 4B,C) and observed 3,895,502 (∼87%) and 582,084 (∼13%) differential interactions of HT29 *versus* hESC and LoVo *versus* hESC cell lines, respectively (Figure 4A). Differential interactions between two bait fragments (Figures 4D,E) were plotted with interactions within 500 Kb upstream and downstream from the bait, passing the threshold of 5 (threshold >5). The threshold criteria >5 was set up by the Chicdiff (Cairns et al., 2019) tool for significant differential interactions. Similar to the differential interaction analysis, we performed differential RNA-seq analysis using CRC datasets obtained from TCGA databases (The Cancer Genome Atlas Network, et al., 2012). The identified differentially expressed (DE) genes are depicted in Figure 4F. We found approximately twenty-eight thousand transcripts that were differentially expressed (false discovery rate ≤ 0.05). Nearly 62% of transcripts were expressed in cancer cells compared to those in normal cells. In cancer cells, 35% were protein-coding genes, and the remaining were non-coding genes (lincRNA, pseudogenes, antisense, miRNA, etc.). All the transcripts, along with their annotations, are provided in Supplementary Table S3. Gene expressions related to colorectal cancer (primary tumor and metastatic) were compared with blood-derived normal, solid tissue to determine the DE genes using edgeR (Robinson et al., 2010). For the extraction of DE genes that lie within the differential interaction region, we overlapped the genomic coordinates of differential bait fragments with the genomic coordinates of DE genes. In hESC/HT29 and hESC/LoVo case studies, we found 24,438 and 17,385 DE transcripts, respectively, after overlapping. The abovementioned steps enabled finding the repertoire of DE genes due to structural changes at the chromatin loop level.

### 3.3 Disruption in TAD dynamics of HT29 and LoVo cell lines, compared to that in hESCs, involves a subset of colorectal-specific genes

**FIGURE 5:**
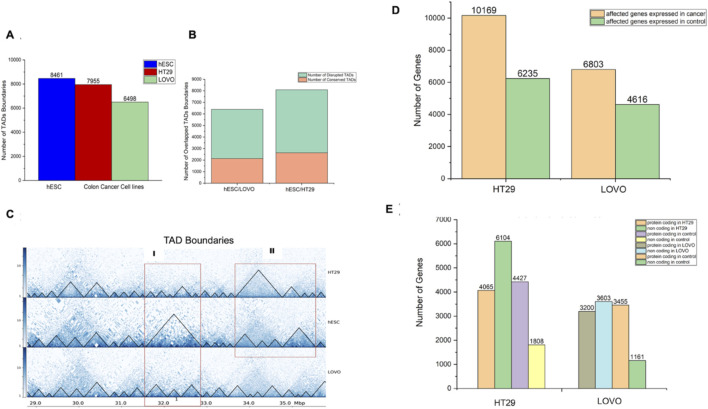
Distribution of genome-wide interactions between CRC cell lines (HT29 and LoVo) and embryonic stem cell line (hESC). Here, TAD refers to intra-chromosomal interaction between two different genomic fragments. **(A)** Number of TAD boundaries for each cell line generated by HiCExplorer (Wolff et al., 2020; Ramírez et al., 2018; Wolff et al., 2018). **(B)** Number of conserved and disrupted TADs between the two cell lines. **(C)** TAD plot of chr1:28788062–35788062 of HT29, hESC, and LoVo cell lines, and red rectangle highlights a major TAD shift of HT29 and LoVo compared to hESCs. **(D)** Affected genes due to shifting of TAD boundaries in HT29 and LoVo cell lines compared with hESCs. **(E)** Numbers of protein-coding and non-coding genes in HT29 and LoVo TADs compared with those in hESC TADs.

In the previous section, we overlaid differentially expressed genes with the bait fragment’s coordinates of the differential interaction region. To understand the distribution of TAD boundary regions with respect to bait fragments, we further overlapped the bait fragments in such a way that the starting bait should lie within the range of TAD boundary regions. Using HiCExplorer, we plotted the TADs with 10 Kb resolution and compared the TAD boundaries among the three cell lines (hESC, HT29, and LoVo). We found 8,461, 7,955, and 6,498 TAD boundaries, respectively, in hESC, HT29, and LoVo cells (Figure 5A). Next, the overlapped TAD boundaries between two cell lines containing at least one DE gene were classified into two categories: conserved TADs and disrupted TADs. The total number of conserved and disrupted TADs (Figure 5B) of hESC/HT29 and hESC/LoVo are 2,637 and 5,448 and 2,138 and 4,255, respectively. The criterion for selecting conserved TADs is that the genes within the overlapping TADs are the same as those in their corresponding TADs. Hence, overlapping TADs that are not conserved were considered disrupted TADs. We then investigated nearby genes in detail around the TAD disrupted regions. We filtered genes based on their position in the TAD boundary shift loci between normal and cancer cells (Figure 5C). We mapped and filtered the disrupted genes in TAD shifting boundary regions with the TCGA database (Figure 5D). We found that 47% of the disrupted genes were protein-coding and 53% were non-coding genes, which included lincRNAs, miRNAs, pseudogenes, and other non-coding variants in the hESC/LoVo cells. Similarly, 40% expressed genes were protein-coding and 60% were non-coding genes in hESC/HT29 cells (Figure 5E). This approach allowed us to monitor the distribution of nearby genes across TAD boundaries. Taken together, these findings provided insights into the role of regulatory mechanisms in structural instability in CRC compared to normal cells.

### 3.4 Gene ontology, network analysis, and mutational study of colorectal-specific genes in the vicinity of disrupted TAD boundaries

**FIGURE 6:**
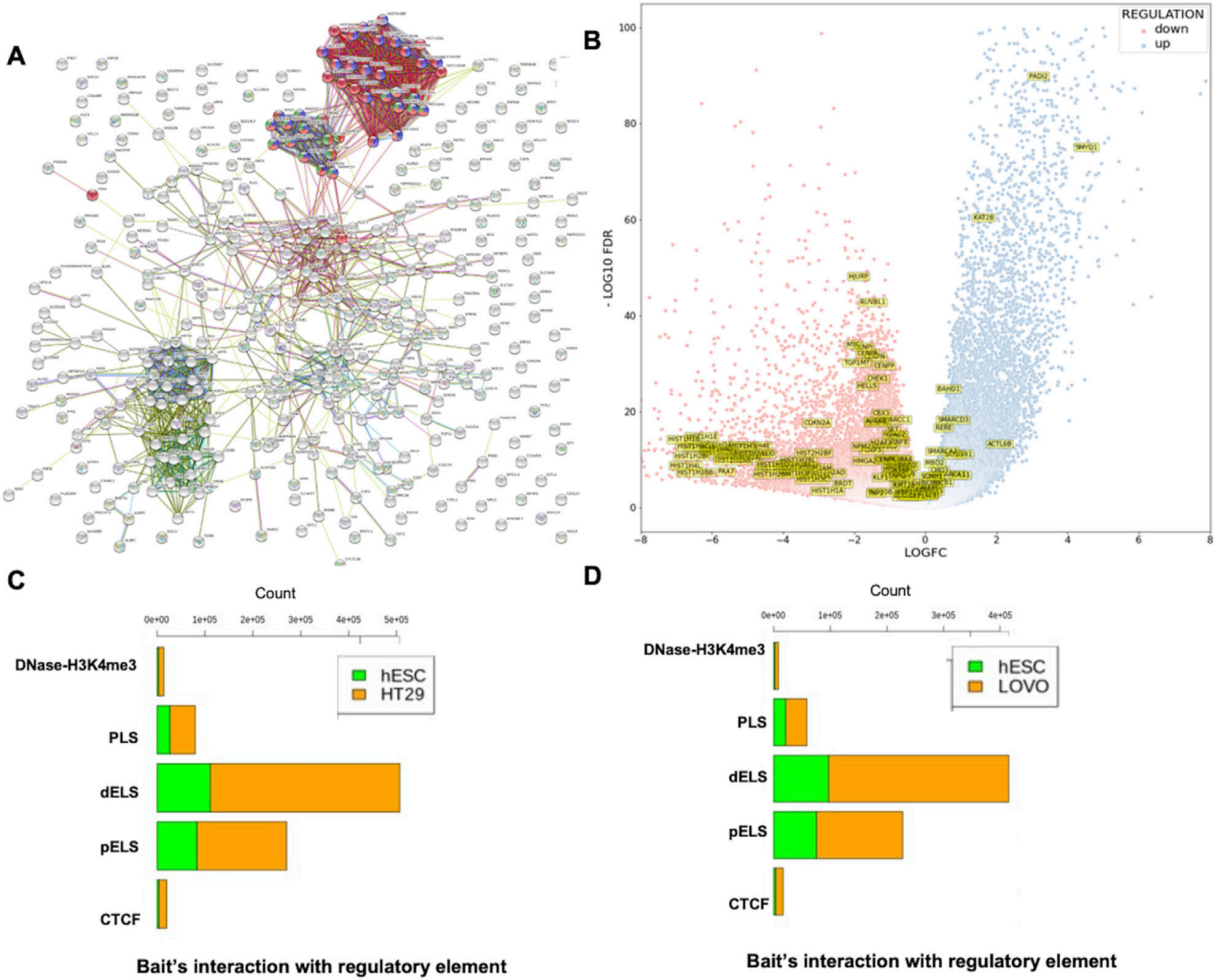
Functional annotation of genes extracted from TAD disruption regions and proportion of cis-regulatory interaction with bait fragments. **(A)** Network visualization of genes and gene families associated with chromatin remodeling (von Mering et al., 2003) (green), chromatin assembly (blue), and chromatin organization (red). **(B)** Volcano plot showing the expression pattern of chromatin remodeling, chromatin assembly, and chromatin organization associated genes that had been extracted from Gene Ontology analysis. **(C)** Total number of bait interactions with regulatory elements (promoter-like (PLS), proximal enhancer-like (pELS), distal enhancer-like (dELS), DNase-H3K4me3, and CTCF regions in disrupted TAD regions of hESC and HT29 cell lines in hESC/HT29. **(D)** Total number of bait interactions with regulatory elements (promoter-like (PLS), proximal enhancer-like (pELS), distal enhancer-like (dELS), DNase-H3K4me3, and CTCF regions in disrupted TAD regions of hESC and LoVo cell lines in hESC/LoVo.

Functional analysis revealed that most of the genes found at the disrupted TAD boundaries were involved in chromatin remodeling, chromatin assembly, and chromatin organization (Table 1). Network analysis revealed that SWI/SNF regulatory networks and histone family clusters were significantly associated with chromatin remodeling, chromatin assembly, and chromatin organization (Figure 6A). The results of enriched Gene Ontology and pathway analyses for both HT29 and LoVo cell lines are provided in Supplementary Table S4. Figure 6B depicts a volcano plot showing the list of the affected genes that were involved in chromatin remodeling, chromatin assembly, and chromatin organization expressed in cancer cells. The heatmap and normalized count of the list of affected genes are listed in Supplementary Figure S1. Furthermore, we integrated the mutational datasets with the affected genes due to TAD changes and examined those affected genes that were flagged as oncogenes in the CRC and COSMIC databases (Tate et al., 2019; Chisanga et al., 2016). Mutational analysis revealed that AR1D1A, ATRX, centromere protein complex, histone complex, chromatin organizers SATB1 and SATB2, SWI1/SNF1 complex, and histone deacetylases were frequently mutated in this dataset. Approximately 585 genes exhibited mutations, specifically in LoVo and HT29 cells, in which nonsynonymous and missense mutations were prominent. Supplementary Figures S2A, B provide the frequency of mutations in terms of CDs and nucleotide changes in genes associated with chromatin-associated genes collected from Gene Ontology analysis, which lie in TAD disruption regions according to Tate et al. (2019) and Chisanga et al. (2016). Supplementary Figures S2C, E list the various types of mutations in genes collected from the database (Chisanga et al., 2016), which lie in the TAD disruption regions. We performed a comparative analysis with the list of chromatin remodeling-associated genes with relevant literature related to CRC (Chisanga et al., 2016; Chubb et al., 2016; Rudd et al., 2005). We obtained 33, 41, and 106 common genes when comparing our study with the previous CRC studies (Chisanga et al., 2016; Chubb et al., 2016; Rudd et al., 2005), respectively (Supplementary Figure S3). Finally, we obtained 10 unique genes that were commonly affected in the cited CRC studies (Chisanga et al., 2016; Chubb et al., 2016; Rudd et al., 2005) and in our study. These genes are TOP1MT, HELLS, AURKB, CHEK1, SMARCC1, TTF1, HDAC1, SCMH1, RERE, and PADI2, out of which HDAC1, RERE, and PADI2 are known oncogenes (Liu et al., 2017) (Supplementary Figure S3). These findings suggest that the shifting of TAD boundaries affects gene regulation in CRC.

**TABLE 1:** List of genes associated with chromosome organizations and assembly.

Function	Name of the genes
Chromatin assembly	Histone H1 HIST1H1A, HIST1H1B, HIST1H1D, and HIST1H1E Histone H2 HIST1H2BB, HIST1H2BE, HIST1H2BF, HIST1H2BI, HIST1H2BL, HIST1H2BM, and HIST1H2BO Histone H3 HIST1H3A, HIST1H3B, HIST1H3C, HIST1H3F, HIST1H3I, and HIST1H3J Histone H4 HIST1H4A, HIST1H4B, HIST1H4C, HIST1H4D, HIST1H4E, HIST1H4L, and HIST2H2BF
Regulation of chromatin remodeling	CDKN2A, HIST1H4A, HIST1H4B, HIST1H4C, HIST1H4D, HIST1H4E, and HIST1H4L INO80, SMARCA1, SMARCA2, SMARCA4, SMARCA5, SMARCB1, SMARCC1, SMARCC2, SMARCD1, SMARCD2, and SMARCD3
Chromatin organization and nucleosome organization	HIST1H2AB, HIST1H2AD, HIST1H2AH, HIST1H2AJ, HIST1H2AL, HIST1H2AM, HIST1H3A, HIST1H3B, HIST1H3C, HIST1H3F, HIST1H3I, HIST1H3J, HIST1H4A, HIST1H4B, HIST1H4C, HIST1H4D, HIST1H4E, HIST1H4L, and HIST2H2AB CDKN2A, CENPN, CENPU, CENPT, HDAC2, CENPW, CENPQ, CENPI, H2AFX, CHEK1, CBX3, TNP2, CASC5, AURKB, ITGB3BP, RBBP7, SET, CHRAC1, SUPT16H, ATRX, HJURP, ACTB, ACTL6B, ARID1A, BAHD1, BAZ1B, BAZ2A, BRD4, BRDT, CBX3, CENPA, CENPK, CENPO, CENPP, CHD4, CHRAC1, DMAP1, ESR1, FOXP3, HDAC1, HDAC2, HDAC5, HELLS, HILS1, HMGA2, HNRNPC, INO80E, INO80C, INO80B, INO80B-WBP1, KAT2B, KLF1, KMT2B, MBD2, MEN1, MIS18A, MIS18BP1, MYC, NASP, NPM2, OIP5, PADI2, PAX7, PBRM1, PSME4, RBBP7, RERE, RNF8, RUVBL1, RUVBL2, SATB1, SATB2, SCMH1, SIRT1, SMYD1, TOP1MT, and TTF1

### 3.5 Distribution of the captured promoters’ interactions with candidate cis-regulatory elements derived from ENCODE data

As mentioned above, we used CHiCAGO, a PCHi-C interaction tool, to identify bait interactions with other-end genomic loci using PCHi-C data (Figure 3). We only selected bait IDs having a non-zero bait count and bait count differences greater than or equal to 10. Following the abovementioned criteria, we obtained 115,198 and 630,095 bait interactions with other end (OE) regions of hESC and HT29 in the hESC/HT29 sample (Supplementary Figure S4A). Similarly, 112,383 and 554,536 bait interactions with OE regions of hESC and LoVo in the hESC/LoVo sample (Supplementary Figure S4B). For the sake of convenience, we referred to the above process as STAGE 1. Again, we filtered the bait IDs from STAGE1 that had common bait IDs generated in the previous section (Figure 4A). We referred to this filtered interaction as STAGE 2. Finally, we filtered bait IDs of STAGE 2 that have common bait IDs with those of the affected genes due to TAD disruption (Figure 5B). The bait IDs of the list of affected genes can be filtered from bait map IDs. The final bait interactions file is referred to as STAGE 3. We overlapped the OE region of STAGE 3 interaction files with regulatory elements such as promoter-like (PLS), proximal enhancer-like (pELS), distal enhancer-like (dELS), DNase-H3K4me3, and CTCF regions (https://screen-v2.wenglab.org) (ENCODE Project Consortium et al., 2020). The stage-wise details of the total number of bait interactions with regulatory elements of hESC/HT29 and hESC/LoVo are tabulated in Supplementary Table S5. It is clearly evident that there are high proximal enhancer-like (pELS) and distal enhancer-like (dELS) interactions with promoter-capture counts, compared to those with the remaining cis-regulatory interactions (Figures 6C,D).

### 3.6 Role of enhancer–promoter interactions within TAD disruption regions in identifying disease-relevant target genes in CRC

**FIGURE 7:**
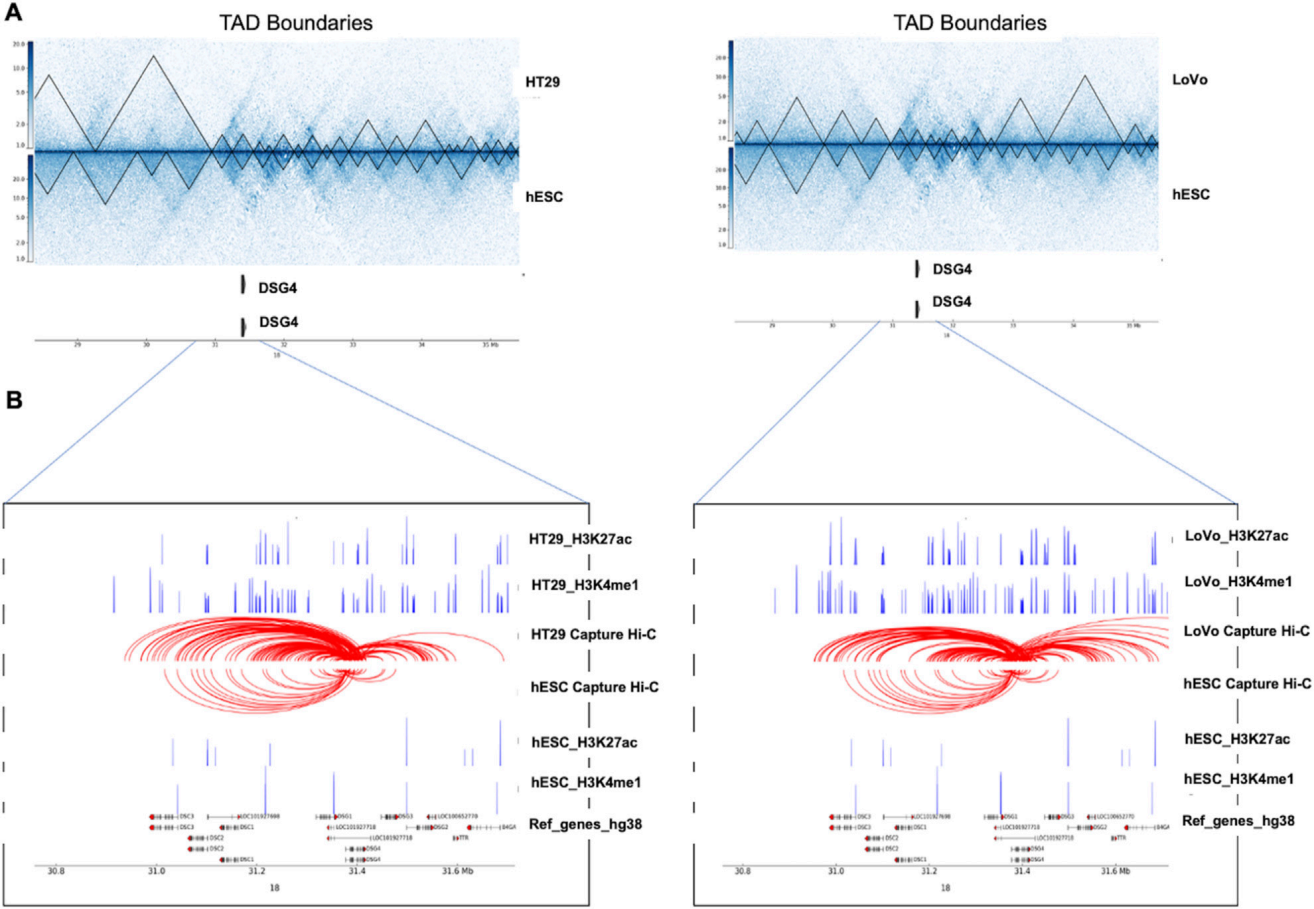
Impact of structural changes on gene regulation between cancer cell lines (HT29 and LoVo) and normal cell lines (hESCs). **(A)** The ∼ 7 Mb region of chromosome 18 encompassing the *DSG4* gene is shown along with TAD boundaries of Hi-C interaction maps at 10 Kb resolution for case (HT29 and LoVo) and control (hESC). **(B)** Zoomed-in view of the *DSG4* locus in case (HT29 and LoVo) and control (hESC) along with corresponding PCHi-C interactions and ChIP-seq data for H3K27ac and H3K4me1 are displayed in blue peaks. Filtered DSG4 read counts used by CHiCAGO are displayed in red, with the corresponding significant interactions shown as arcs. For clarity, only DSG4 interactions were shown.

The integration of PCHi-C with Hi-C was performed to explore the genomic landscape from the loop level to the TAD-level. This was then combined with differentially expressed genes from the TCGA database and from HT29 (case) *versus* NT2D1 (control) cell lines was to understand gene regulation. We also overlapped these data with histone modification marks (H3K4me1 and H3K27ac) to verify the promoter interaction with the enhancer, which overall mimics the impact of TAD disruption on a gene’s expression. These could serve as key genes for potential therapeutic targets. Based on the capture count and log2fold value, we selected a few statistically significant DE genes that lie in the TAD disruption regions. The statistically significant gene list includes long non-coding genes (MALAT1, NEAT1, FTX, and PVT1), small nucleolar genes (SNORA26 and SNORA71A), and protein-coding genes (TMPRSS11D, TSPEAR, and DSG4). In this study, statistical significance refers to selecting the top-hit genes based on high capture counts, which have enhancer–promoter loops, lie within TAD disruption regions, and are upregulated genes in CRC. For the visualization of Hi-C and PCHi-C interaction maps in the context of gene regulation, we highlighted one of the statistically significant upregulated gene loci, *DSG4*, in Figures 7A,B, with the remaining loci in Supplementary Figure S5-11. The *DSG4* gene lies within the TAD disruption region in case cells (HT29 and LoVo) compared to control cells (hESCs), as identified by Hi-C analysis. Similarly, PCHi-C detected a higher frequency of interactions between the *DSG4* promoter and histone modification marks H3K27ac and H3K4me1 in case cells (HT29 and LoVo) compared to the control cells (hESCs). TAD-based analysis helps in defining a gene’s cis-regulatory landscape, while high-resolution promoter interaction data provide the necessary precision for accurately mapping the enhancer–promoter interactions. There were more active enhancer marks and enhancer–promoter loop formations in the case cells than in the control cells in the vicinity of the *DSG4* gene locus, suggesting its upregulation (Figures 7A,B). PCHi-C techniques employed across various cell types detected numerous enhancer–promoter interactions, uncovering significant differences in promoter structure among these cell types and during differentiation (Schoenfelder et al., 2015; Mifsud et al., 2015; Javierre et al., 2016; Freire-Pritchett et al., 2017; Rubin et al., 2017; Siersbæk et al., 2017; Montefiori et al., 2018). The variation in enhancer–promoter interactions within TAD disruption regions (Figures 7A,B) suggests that disease-relevant cell types (such as HT29 and LoVo) serve as tractable models for effectively probing the gene regulatory mechanisms associated with disease loci.

### 3.7 Validation of the dysregulated genes revealed putative biomarkers

**FIGURE 8:**
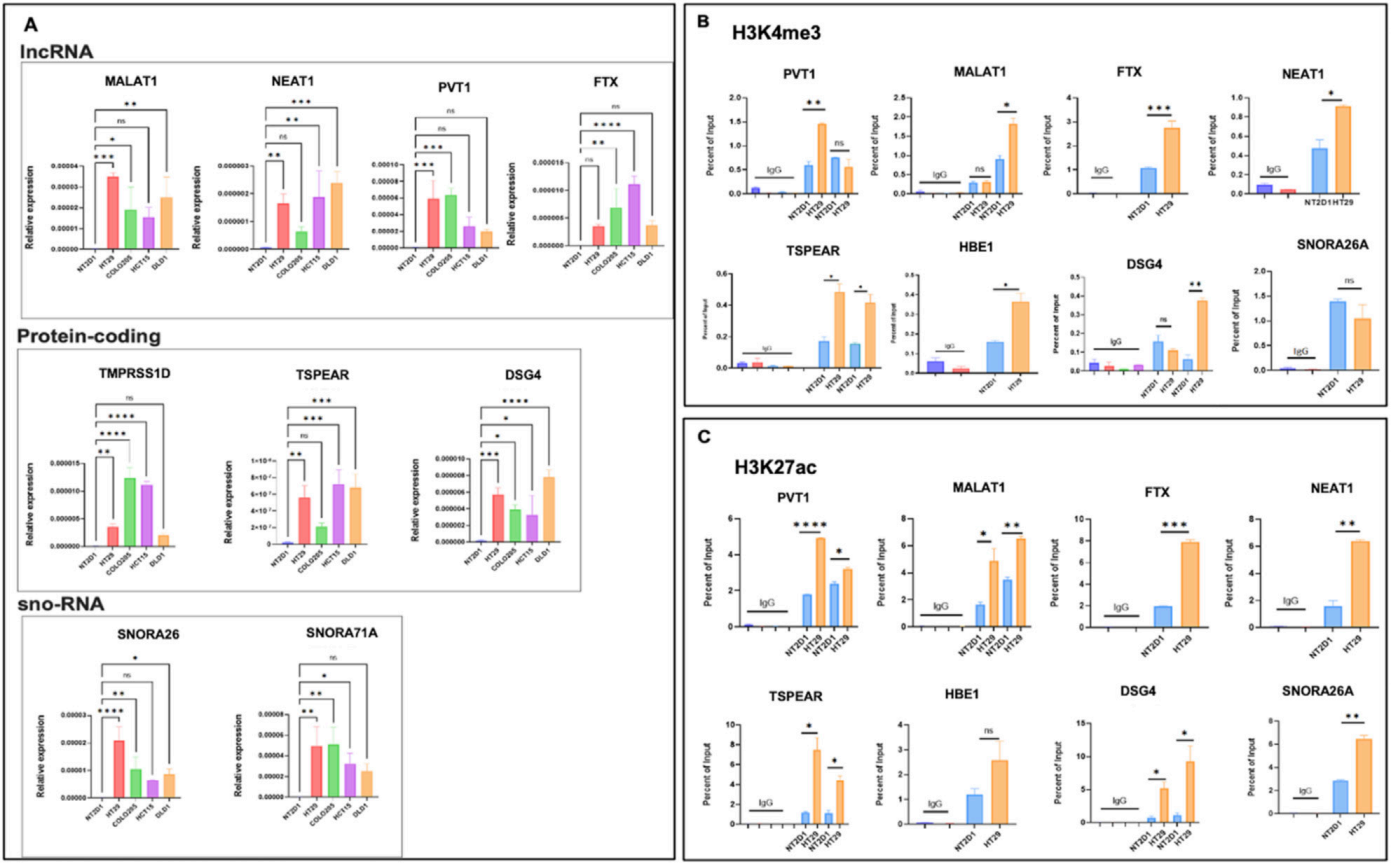
Validation of target genes extracted from integrative analysis. **(A)** qRT-PCR analysis of target genes in NT2D1, HT29, Colo205, HCT15, and DLD1 cell lines, showing relative enrichment of gene expression in the CRC cell lines compared to NT2D1 cells. **(B, C)** ChIP-qPCR for H3K4me3 and H3K27ac modifications, respectively, on the transcription start site (TSS) upstream regions of target genes. The cell lines used are indicated below the bars. Three biological replicates were used for statistical analysis using GraphPad v9.1. A one-way ANOVA or unpaired t-test was used as the significance test. *p < 0.05, **p < 0.01, ***p < 0.001, ****p < 0.0001, and ns,non-significant.

As discussed above, using our combined Hi-C, promoter-capture Hi-C, RNA-seq, and ChIP-seq analysis, we have uncovered new potential targets that exhibit a high degree of dysregulation in CRC datasets with respect to the 3D structure. To validate whether the predictions of our analysis hold true, we next sought to monitor the expression profiles of a few of the statistically significant hits from our dataset in CRC models, some of which were previously reported to be dysregulated in CRC. lncRNA PVT1 has been implicated in the progression of colorectal cancer via the VEGFA-AKT axis (Wu et al., 2020). lncRNA NEAT1 modulates chromatin accessibility in CRC and directly regulates Myc and ALDH1 (Zhu et al., 2020). Similarly, recent studies (Zhao et al., 2020; Yang et al., 2018; Jia et al., 2022; Guo et al., 2015) have reported that lncRNA FTX plays a crucial role in the initiation and progression of CRC. We used a highly malignant colorectal cell line HT29 and compared it to an embryonic cell line NT2D1 in our experimental validations to closely represent the primary datasets used for the bioinformatics analysis. We selected our targets, in which the contact frequency was at least two-fold. We observed that expression levels of genes belonging to long non-coding genes (MALAT1, NEAT1, FTX, and PVT1), small nucleolar genes (SNORA26 and SNORA71A), and protein-coding families (TMPRSS11D, TSPEAR, and DSG4) were significantly higher in HT29 and various other colorectal model cell lines than in NT2D1 embryonic cells (Figure 8A). Next, we monitored the relative levels of transcriptional activation-associated chromatin modifications H3K4me3 and H3K27ac at the promoters of these genes by performing ChIP-quantitative PCRs. We observed a significant enrichment of both these modifications at the promoters of all selected target genes (Figures 8B,C), suggesting a hierarchical modification of the chromatin following TAD disruption. Collectively, these analyses suggest that changes in transcription-associated chromatin modifications correlated with the 3D chromatin changes shown previously, leading to the activation of the transcription machinery.

The integrative analysis reported above is based on bulk-seq data. In bulk-seq studies, samples are treated as a homogeneous population. However, the cell populations in the human body are heterogeneous in nature, and each cell reflects unique activity. Due to recent breakthroughs, it is now possible to analyze the transcriptome at the single-cell level for millions of cells (Stuart and Satija, 2019; Khaliq et al., 2022). This enabled us to differentiate, characterize, and classify each cell at the transcriptome level, which leads to the prediction of rare cell populations. Taking advantage of the single-cell human colon cancer atlas database (Pelka et al., 2021), we analyzed transcriptionally profiled 371,223 tumor and adjacent normal cells generated based on scRNA-seq. A detailed analysis is provided in Supplementary Material M1. The purpose of the single-cell RNA-seq analysis is to assess the enrichment of the potential targeted genes (MALAT1, NEAT1, FTX, PVT1, SNORA26, SNORA71A, TMPRSS11D, TSPEAR, and DSG4) at the single-cell level. We did not observe a significant enrichment score at the single-cell level despite these genes exhibiting a high degree of dysregulation at the bulk-seq level.

## 4 Discussion

TADs are increasingly recognized as pivotal elements of 3D chromosome organization and gene regulation. Our objective was to understand the communication between enhancer-promoter interactions and impact on gene expression. In this study, we investigated how the abnormalities in 3D chromosome folding affect gene expression patterns in colorectal cancer. The instability of cancer genomes often results in a higher frequency of TAD disruption in cancer cells compared to their normal counterparts. Such disruptions could trigger significant alterations in gene expression, potentially contributing to the initiation and progression of tumorigenesis. Our comprehensive analysis attempted to explore the molecular basis of the genetic risk for CRC. The configurations of TADs in cancer cells could thus offer valuable insights into the genetic underpinnings of gene expression in these cells, potentially identifying specific targets for therapeutic interventions. In our study, we focused on identifying multiple potential therapeutic targets for colorectal cancer by systematically integrating 3D datasets with 1D datasets. We employed publicly available datasets including Hi-C, PCHi-C, ChIP-seq, RNA-seq, and single-cell RNA-seq data towards this comprehensive analysis. Generating high-resolution Hi-C data through Hi-C experiments requires the use of millions of mammalian cells, resulting in the generation of billions of paired-end reads. Because of the substantial costs of sequencing, most Hi-C datasets exhibit a relatively low resolution, which might not be suitable for examining detailed interactions in the vicinity of regions of interest, such as disease-associated loci.

To overcome this limitation, we integrated PCHi-C data with Hi-C to understand the dynamics of regulatory chromosomal interactions from local-to-TAD level. This approach enabled us to investigate the structural organization and changes in chromatin interactions across different cellular contexts. During our analysis, we observed that most interactions were found in cancer cell lines (HT29, LoVo) compared to hESCs. This finding suggests a higher frequency of structural alterations in tumor cells, highlighting the extensive chromatin remodeling and genomic instability associated with tumor cell population in contrast to human embryonic stem cells. Such modifications may contribute to cancer-specific transcriptional programs by reshaping regulatory interactions.

**FIGURE 9:**
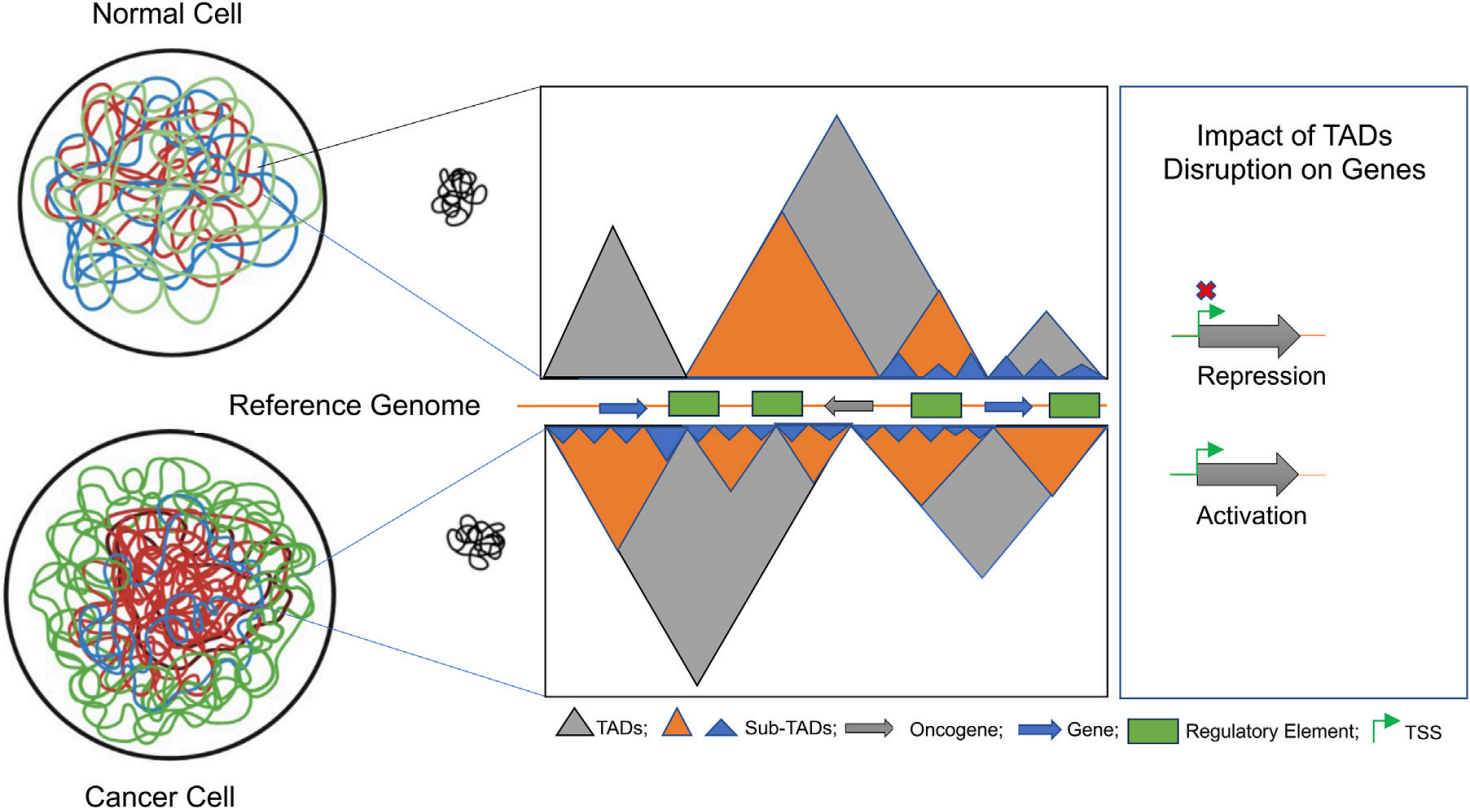
Schematic depiction of the architectural changes in TADs during tumorigenesis: we explored how dynamic changes in the 3D genome architecture influence gene regulation in colorectal cancer (CRC). Fine-scale 3D-genome interactions, combined with their association with epigenomic marks, were analyzed to identify novel biomarkers for CRC progression. The disruption of TAD dynamics was found to involve a subset of colorectal-specific genes, as depicted schematically.

TADs exhibit evolutionary conservation and serve as crucial elements in governing and facilitating long-range regulation of gene expression_67_. They act as self-contained domains that confine enhancer-promoter interactions, ensuring precise gene expression control. When a TAD boundary is deleted, it can lead to the fusion of two adjacent TADs, resulting in the misregulation of genes previously restricted to separate regulatory environments. Genomic rearrangements that fragment existing TADs and create novel regulatory domains, while preserving the original TAD boundaries. The disruption of TAD boundaries can lead to abnormal gene expression patterns by exposing genes to inappropriate regulatory elements. This disruption is frequently observed in cancer cells, where alterations in the 3D chromatin architecture drive oncogenic transcriptional programs and plays a crucial role in tumorigenesis_68–70_ (Figure 9). Comprehending the significance of TAD disruption and long-range chromatin interactions is essential for gaining insights into the broader mechanisms of gene regulation. Additionally, it sheds light on how genomic rearrangements and mutations in cancer genomes can result in the atypical expression of oncogenes and tumor suppressors. This knowledge contributes not only to our understanding of gene regulation in a general context but also to our comprehension of the molecular underpinnings of cancer progression.

For gene expression analysis, we performed differential RNA-seq analysis using colorectal cancer samples (primary tumor, metastatic) compared with normal samples (blood-derived normal, solid tissue) obtained from the TCGA databases. We filtered differentially expressed genes found around variably interacting regions to check the distribution of DGEs across TADs boundaries. We used H3K27ac and H3K4me1 histone modification marks for understanding the gene's cis-regulatory landscape to precisely map the enhancer-promoter interactions. The functional study revealed that most of the genes upregulated in cancer were found around TAD disruption regions, and are involved in chromatin remodeling, chromatin assembly and chromatin organization. We also integrated the mutational datasets collected from databases like cosmic database_52,53_ and human oncogene database56 with the affected genes due to TAD disruptions. Results of such analyses revealed that genes encoding key chromatin associated regulators including AR1D1A, ATRX, centromere protein complex, histone complex, SATB1, SATB2, SWI1/SNF1 complex, histone deacetylases were frequently mutated in these datasets.

Finally for experimental validation, we filtered a statistically significant gene-list based on high capture count and log2 fold change in transcript expression; and with relevant literature related to colorectal cancer. We found expression of long non-coding genes (MALAT1, NEAT1, FTX and PVT1), small nucleolar (SNORA26 and SNORA71A) and protein-coding (TMPRSS11D, TSPEAR and DSG4) were significantly much higher in HT29 compared to NT2D1; and enriched with activation-associated histone modifications H3K4me3 and H3K27ac. Our data, while confirming the expression patterns, strongly argues in favor of a priori involvement of epigenomic mechanisms in form of dynamic histone modifications as well as the chromatin interaction changes that modulate the gene expression.

Additionally, for understanding the expression patterns of target genes across diverse cell types, we analyzed 371,223 tumor and adjacent normal cells taken from the single cell human colon cancer atlas database. We found expression of FTX and PVT1 is contributed from the rare cell population while MALAT1 and NEAT1 were expressed almost everywhere in diverse cell populations. However, expression of TSPEAR and DSG4 was not observed in the scRNA-seq analysis. Collectively, our study suggests that changes in 3-D genomic architecture affect transcriptome signatures which might be associated with tumor-suppressive transcriptional programs (Figure 9). Such integrative analysis resulted in identification of multiple target genes which may potentially serve as biomarkers and could be used to better understand CRC progression.

## Data Availability

The dataset analyzed in the study is publicly available. The accession number can be found in the article/Supplementary Material.
