# Supplementary material for: Integrated promoter-capture Hi-C and Hi-C analysis reveals fine-tuned regulation of the 3D chromatin architecture in colorectal cancer
Source: Front Genet. 2025 Mar 28;16:1553469. doi: 10.3389/fgene.2025.1553469 (PMC11985782; doi:10.3389/fgene.2025.1553469)
Supplement: Supplementary file 1 [file DataSheet2.pdf]

Combined promoter-capture Hi-C and Hi-C analysis reveals a fine-tuned regulation of 3D chromatin architecture in colorectal cancer

Ajay Kumar Saw[1], Ayush Madhok[1,&], Anupam Bhattacharya[2,3,&], Soumyadeep Nandi[4,#a,*] and Sanjeev Galande[1,5,*]

[1]Laboratory of chromatin Biology and Epigenetics, Department of Biology, Indian Institute of Science Education and Research, Pune, 411008, India.

[2]Division of Life Sciences, Institute of Advanced Study in Science and Technology, Vigyan Path, Paschim Boragaon, Garchuk, Guwahati, Assam, India.

[3]Department of Molecular Biology and Biotechnology, Cotton University, Panbazar, Guwahati, Assam, India.

[4] Data Sciences and Computational Biology Centre, Amity Institute of Integrative Sciences and Health, Amity University Haryana, Gurugram, Manesar, 122413, Haryana, India.

[5]Centre of Excellence in Epigenetics, Department of Life Sciences, Shiv Nadar University, Gautam Buddha Nagar, Greater Noida, Uttar Pradesh, India.

[&] These authors contributed equally to this work.

[#a]Current address, Umeå Plant Science Centre, Department of Plant Physiology, Umeå University, Sweden

Supplementary Material M1

Expression pattern of statistically significant genes for therapeutic target in single cell human colon cancer atlas

In the analysis step, first we preprocessed data for preventing outlier cells which could influence downstream analysis. We generated a violin plot before and after performing the quality control (Figure M1(A-B)). In the quality control step, we manually selected the threshold level of number of features (transcripts), number of counts against each feature and mitochondrial percentage of cells. Using the processed data, we generated a t-SNE plot (Figure M1C). The t-SNE plot separated the diverse cell population into 36 clusters enabling high-resolution depiction of the cellular diversity and heterogeneity. Here, we were interested in monitoring the expression patterns derived from the integrative bulk-seq study at single cell level across different cell populations. This helped us to gauge the distribution of the expression patterns of targeted genes across diverse cell populations (Figure M1C). In this study we selected a few statistically significant genes (MALAT1, NEAT1, FTX, PVT1, SNORA26, SNORA71A, TMPRSS11D, TSPEAR and DSG4) as potential therapeutic targets in early colorectal cancer detection or prevention. Out of these 9 genes, 3 genes (SNORA26, SNORA71A, TMPRSS11D) were missing in the single cell gene annotation files. Therefore, we monitored the expression pattern of the remaining six genes using the single cell human colon cancer atlas database[1]. FTX and PVT1 exhibit lower expression levels compared to MALAT1 and NEAT1 (Figure M1D). FTX gene showed significant expression only in B cell, T cell, endothelial cell, epithelial cell, macrophage, and monocyte populations. PVT1 gene showed significant expression only in B cell, T cell, and epithelial cell cells population. However, TSPEAR and DSG4 did not exhibit any significant expression level differences among distinct cell populations. Expression profiling at single cell level helped us to monitor the distribution of gene expression across distinct cell types which could be considered as a key factor in the assessment of their potential as a biomarker.

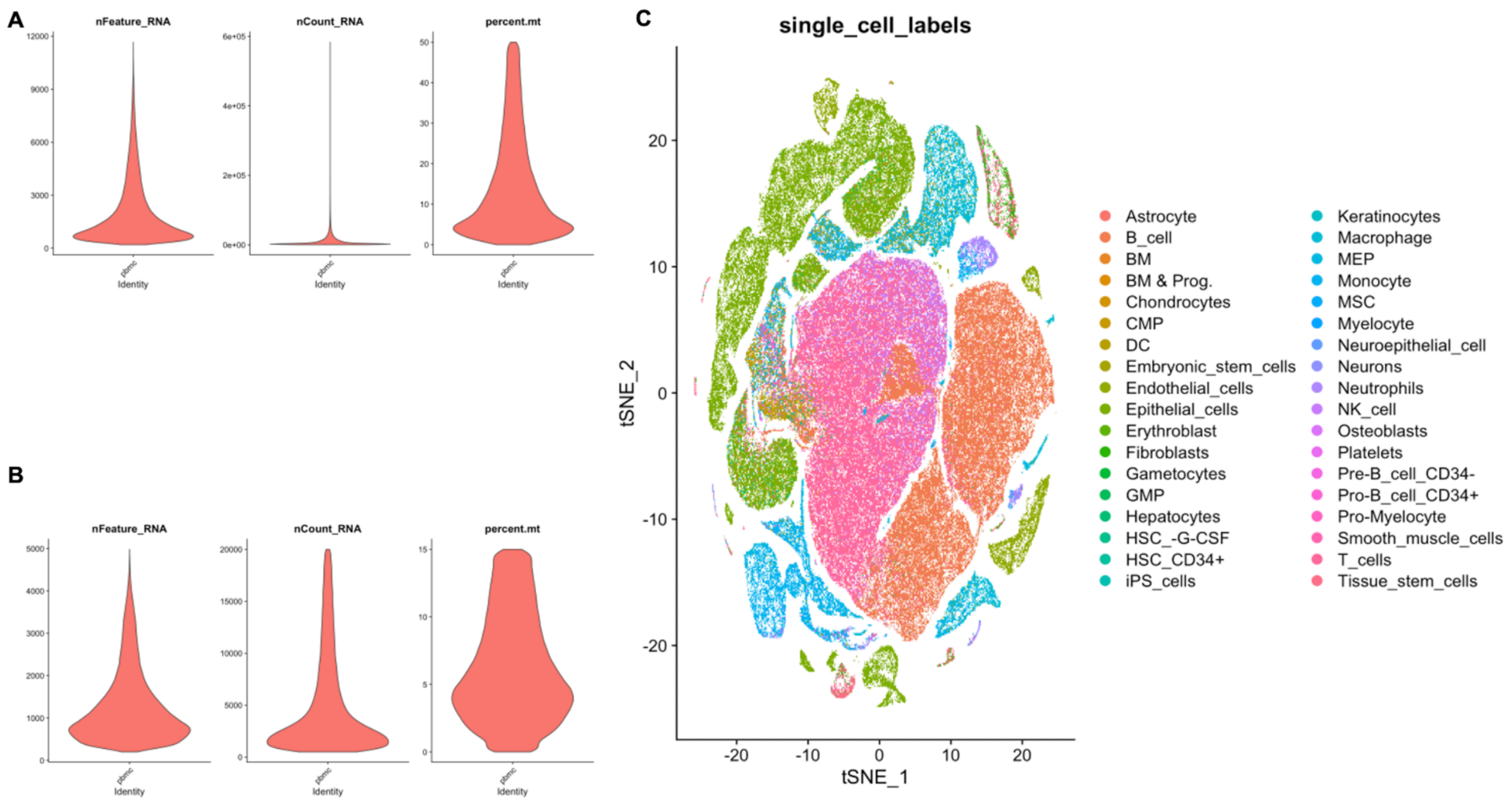
A
nFeature_RNA
nCount_RNA
percent.mt
pbmc
Identity
B
nFeature_RNA
nCount_RNA
percent.mt
pbmc
Identity
C
single_cell_labels
tSNE_1
tSNE_2
Astrocyte
B_cell
BM
BM & Prog.
Chondrocytes
CMP
DC
Embryonic_stem_cells
Endothelial_cells
Epithelial_cells
Erythroblast
Fibroblasts
Gametocytes
GMP
Hepatocytes
HSC_-G-CSF
HSC_CD34+
iPS_cells
Keratinocytes
Macrophage
MEP
Monocyte
MSC
Myelocyte
Neuroepithelial_cell
Neurons
Neutrophils
NK_cell
Osteoblasts
Platelets
Pre-B_cell_CD34-
Pro-B_cell_CD34+
Pro-Myelocyte
Smooth_muscle_cells
T_cells
Tissue_stem_cells

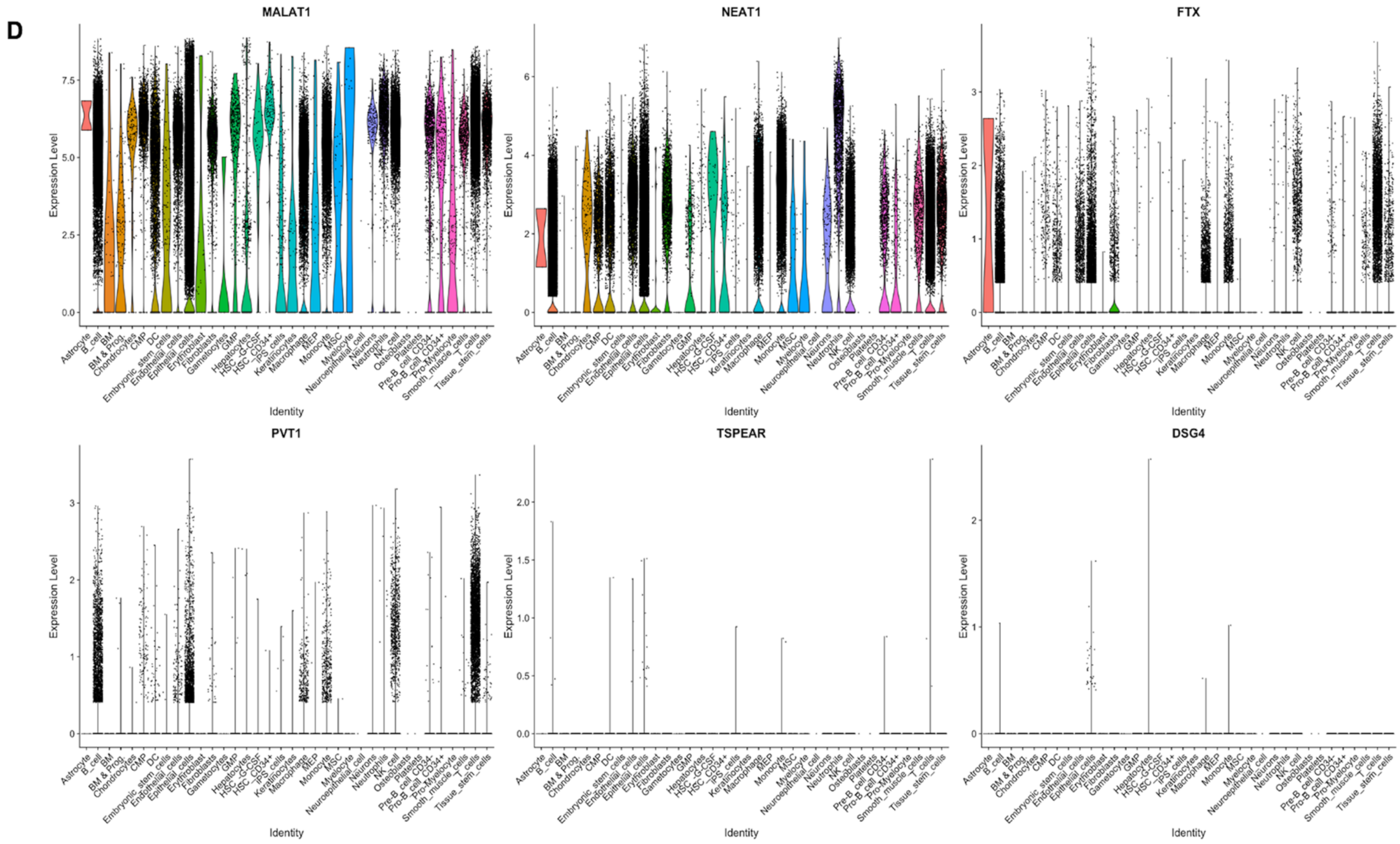

Figure M1. Single-cell RNA-Seq analysis of 371,223 tumor and adjacent normal cells collected from single cell human colon cancer atlas database reveals contributions of target genes across diverse cell populations. (A and B) Violin plot of number of features, number of counts and mitochondrial percentage before and after quality control. (C) t-SNE plot of 371,223 tumor and adjacent normal cells separated the cell population into 36 clusters enabling high-resolution depiction of the cellular diversity and heterogeneity. (D) Violin plots of targeted gene enrichment scores across 36 diverse cell type populations.

Reference

1. Pelka, K. *et al.* Spatially organized multicellular immune hubs in human colorectal cancer. *Cell* **184**, 4734-4752.e20 (2021).
