# Supplementary material for: Integrated promoter-capture Hi-C and Hi-C analysis reveals fine-tuned regulation of the 3D chromatin architecture in colorectal cancer
Source: Front Genet. 2025 Mar 28;16:1553469. doi: 10.3389/fgene.2025.1553469 (PMC11985782; doi:10.3389/fgene.2025.1553469)
Supplement: Supplementary file 3 [file DataSheet4.pdf]

Supplementary Figures

Combined promoter-capture Hi-C and Hi-C analysis reveals a fine-tuned regulation of 3D chromatin architecture in colorectal cancer

Ajay Kumar Saw[1], Ayush Madhok[1,&], Anupam Bhattacharya[2,3,&], Soumyadeep Nandi[4,#a,*] and Sanjeev Galande[1,5,*]

[1]Laboratory of chromatin Biology and Epigenetics, Department of Biology, Indian Institute of Science Education and Research, Pune, 411008, India.

[2]Division of Life Sciences, Institute of Advanced Study in Science and Technology, Vigyan Path, Paschim Boragaon, Garchuk, Guwahati, Assam, India.

[3]Department of Molecular Biology and Biotechnology, Cotton University, Panbazar, Guwahati, Assam, India.

[4] Data Sciences and Computational Biology Centre, Amity Institute of Integrative Sciences and Health, Amity University Haryana, Gurugram, Manesar, 122413, Haryana, India.

[5]Centre of Excellence in Epigenetics, Department of Life Sciences, Shiv Nadar University, Gautam Buddha Nagar, Greater Noida, Uttar Pradesh, India.

[&] These authors contributed equally to this work.

[#a]Current address, Umeå Plant Science Centre, Department of Plant Physiology, Umeå University, Sweden

[*] To whom correspondence should be addressed.

A

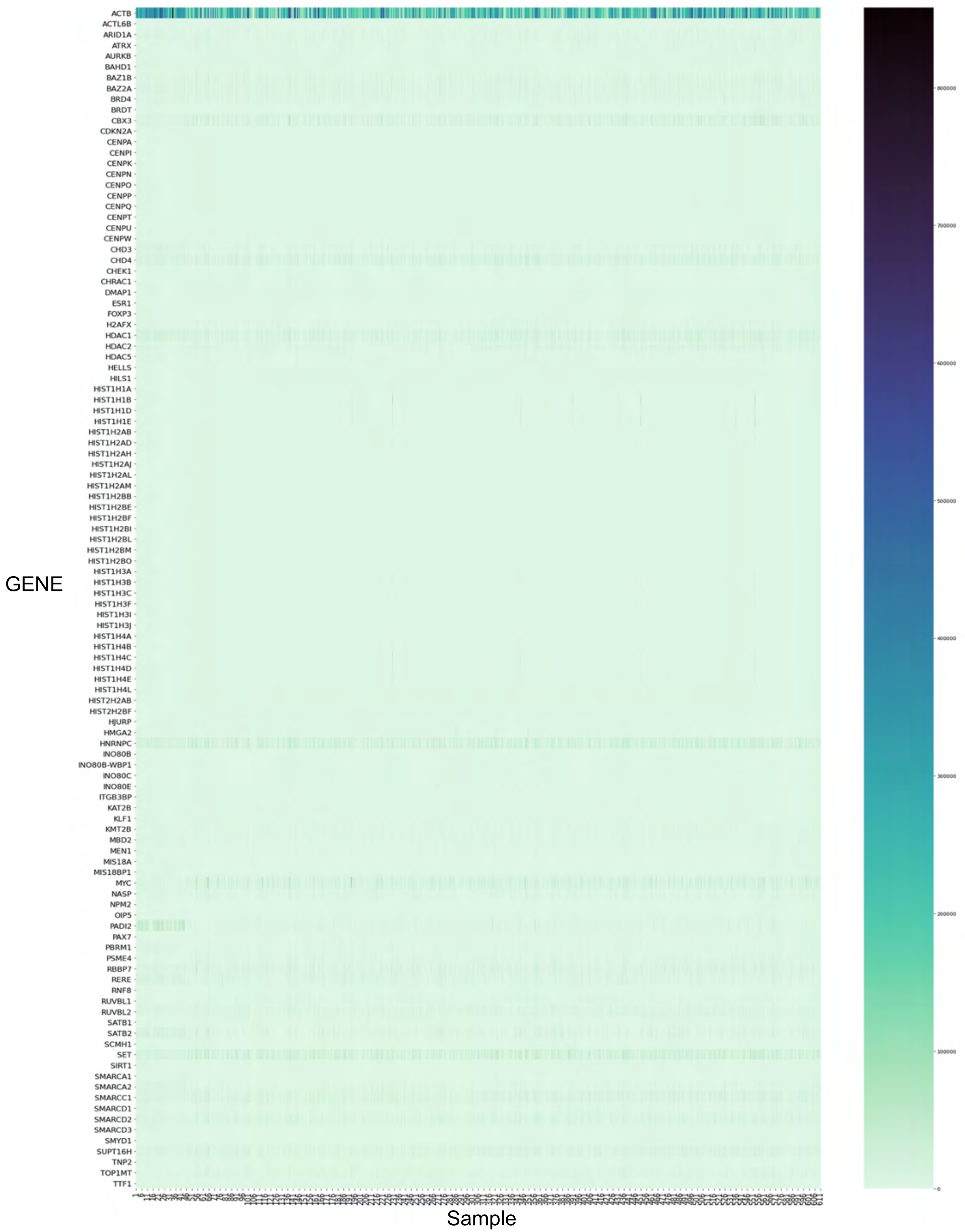

ACTB
ACTL6B
ARID1A
ATRX
AURKB
BAHD1
BAZ1B
BAZ2A
BRD4
BRDT
CBX3
CDKN2A
CENPA
CENPI
CENPK
CENPN
CENPO
CENPP
CENPQ
CENPT
CENPU
CENPW
CHD3
CHD4
CHEK1
CHRAC1
DMAP1
ESR1
FOXP3
H2AFX
HDAC1
HDAC2
HDAC5
HELLS
HILS1
HIST1H1A
HIST1H1B
HIST1H1D
HIST1H1E
HIST1H2AB
HIST1H2AD
HIST1H2AH
HIST1H2AJ
HIST1H2AL
HIST1H2AM
HIST1H2BB
HIST1H2BE
HIST1H2BF
HIST1H2BI
HIST1H2BL
HIST1H2BM
HIST1H2BO
HIST1H3A
HIST1H3B
HIST1H3C
HIST1H3F
HIST1H3I
HIST1H3J
HIST1H4A
HIST1H4B
HIST1H4C
HIST1H4D
HIST1H4E
HIST1H4L
HIST2H2AB
HIST2H2BF
HJURP
HMGA2
HNRNPC
INO80B
INO80B-WBP1
INO80C
INO80E
ITGB3BP
KAT2B
KLF1
KMT2B
MBD2
MEN1
MIS18A
MIS18BP1
MYC
NASP
NPM2
OIP5
PADI2
PAX7
PBRM1
PSME4
RBBP7
RERE
RNF8
RUVBL1
RUVBL2
SATB1
SATB2
SCMH1
SET
SIRT1
SMARCA1
SMARCA2
SMARCC1
SMARCD1
SMARCD2
SMARCD3
SMYD1
SUPT16H
TNP2
TOP1MT
TTF1
GENE
Sample
800000
700000
600000
500000
400000
300000
200000
100000
0

B

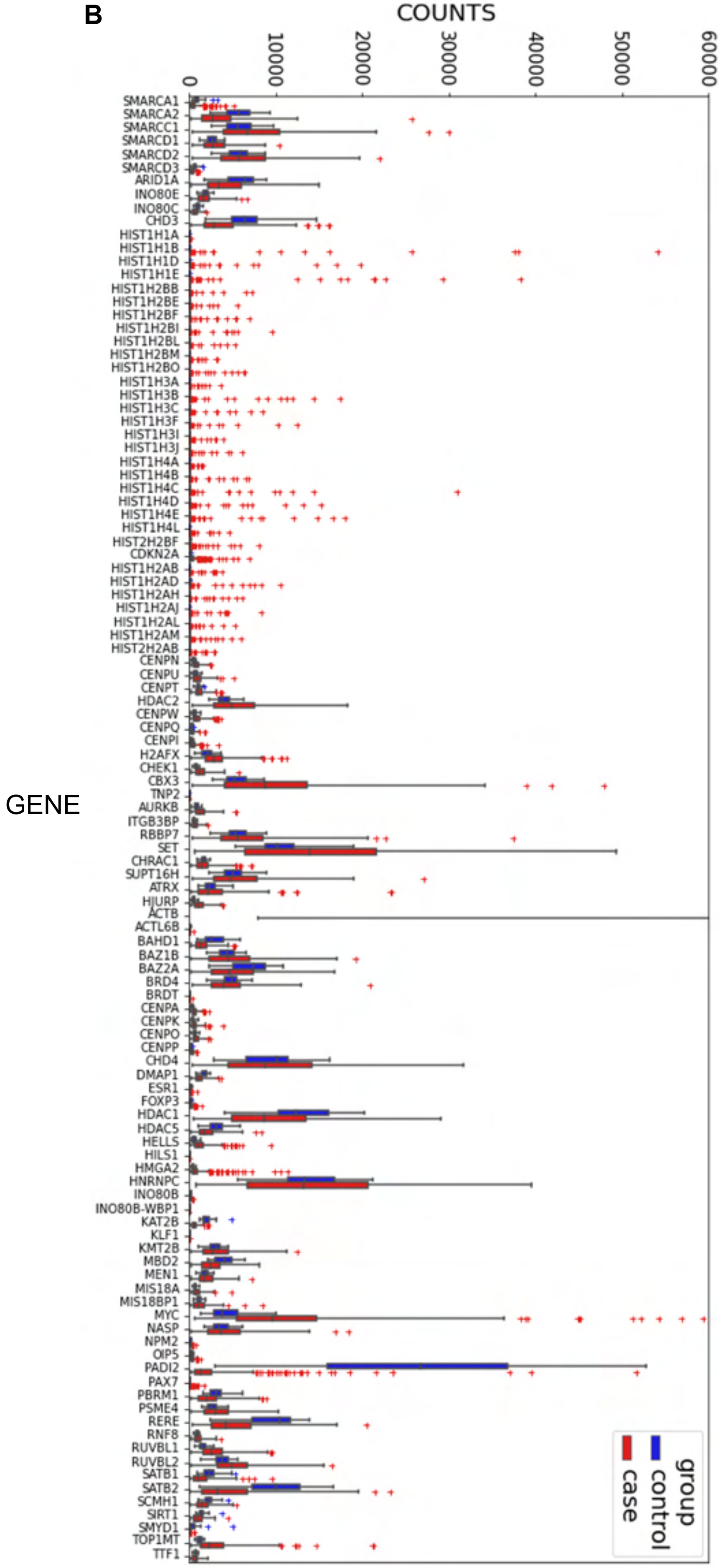

Figure S1. GO ontology analysis. (A) Heatmap and (B) Normalized count showing the expression pattern of chromatin remodeling, chromatin assembly and chromatin organization associated genes which has been extracted from gene ontology analysis.

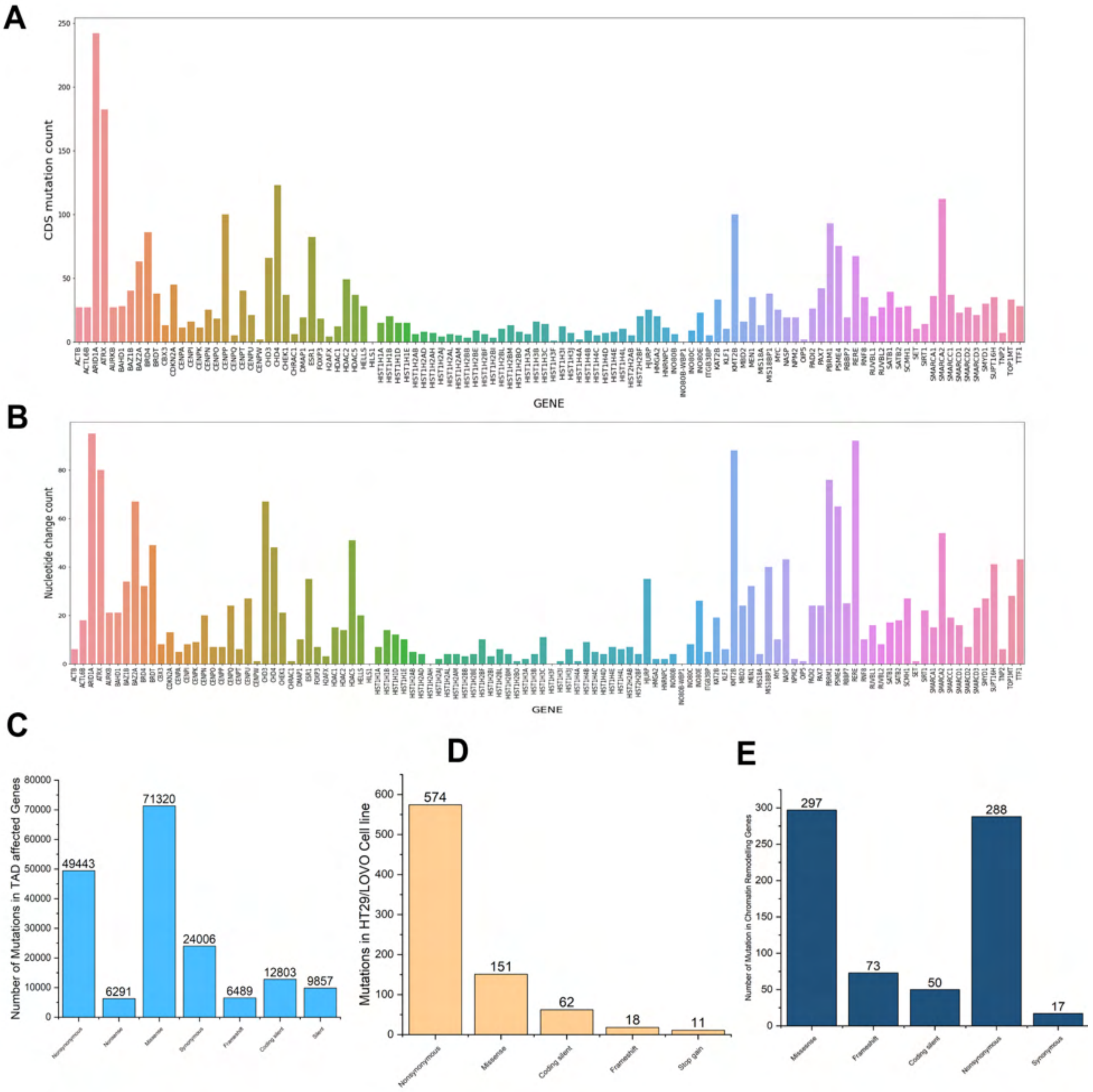

Figure S2. The variation in number and type of mutation. (A) Frequency of CDS mutations in genes associates with chromatin-associated genes collected from gene ontology analysis which lies in TADs disrupted regions[1]. (B) Frequency of mutations of nucleotide changes in chromatin-associated genes collected from gene ontology analysis which lies in TADs disrupted regions[2]. (C) Types of mutations in genes collected from colorectal cancer cell lines database[2] which lies in TADs disrupted regions in our case versus control study. (D) Types of mutations in genes from HT20 and LoVo cell lines[2] lies in TADs disrupted regions in our case versus control study. (E) Type of mutations in chromatin-associated genes collected from gene ontology analysis which lies in TADs disrupted regions[2] .

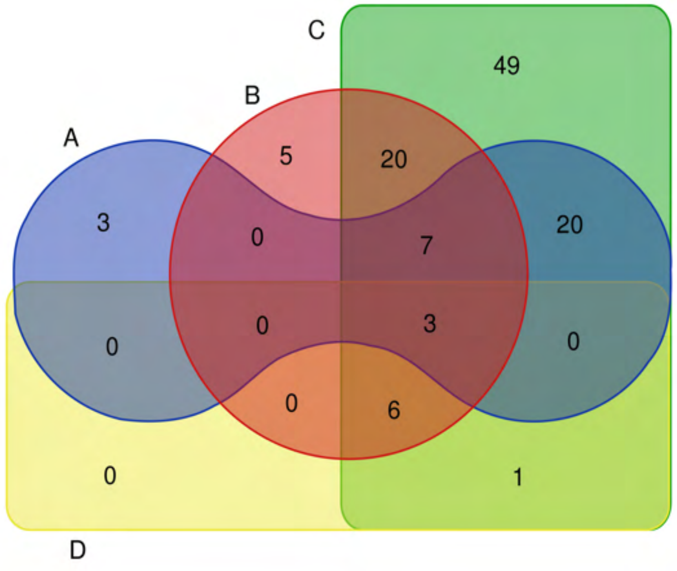

Figure S3. Identification of potential genes in colorectal cancer susceptibility. Venn diagram representing the list of genes in our study which also found in colorectal cancer relevant literature studies[2–5]. Here, number represent distinct gene count and four different colors corresponds to scientific articles[2-5] related to colorectal cancer study.

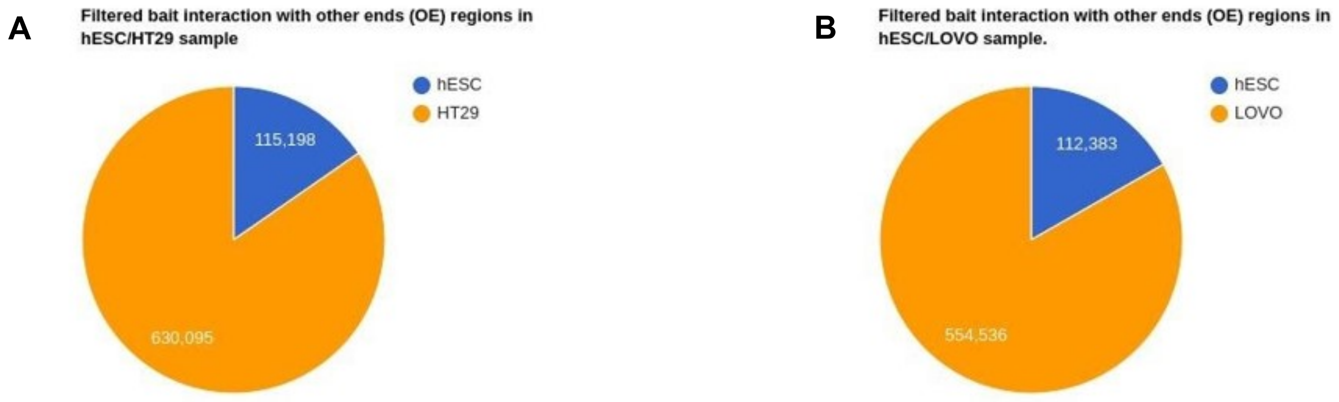

Figure S4. The proportion of capture Hi-C interaction in normal versus cancer cell lines. (A) Filtered bait interaction with other ends (OE) regions of hESC and HT29 in the hESC/HT29 sample. (B) Filtered bait interaction with other ends (OE) regions of hESC and LoVo in the hESC/LoVo sample.

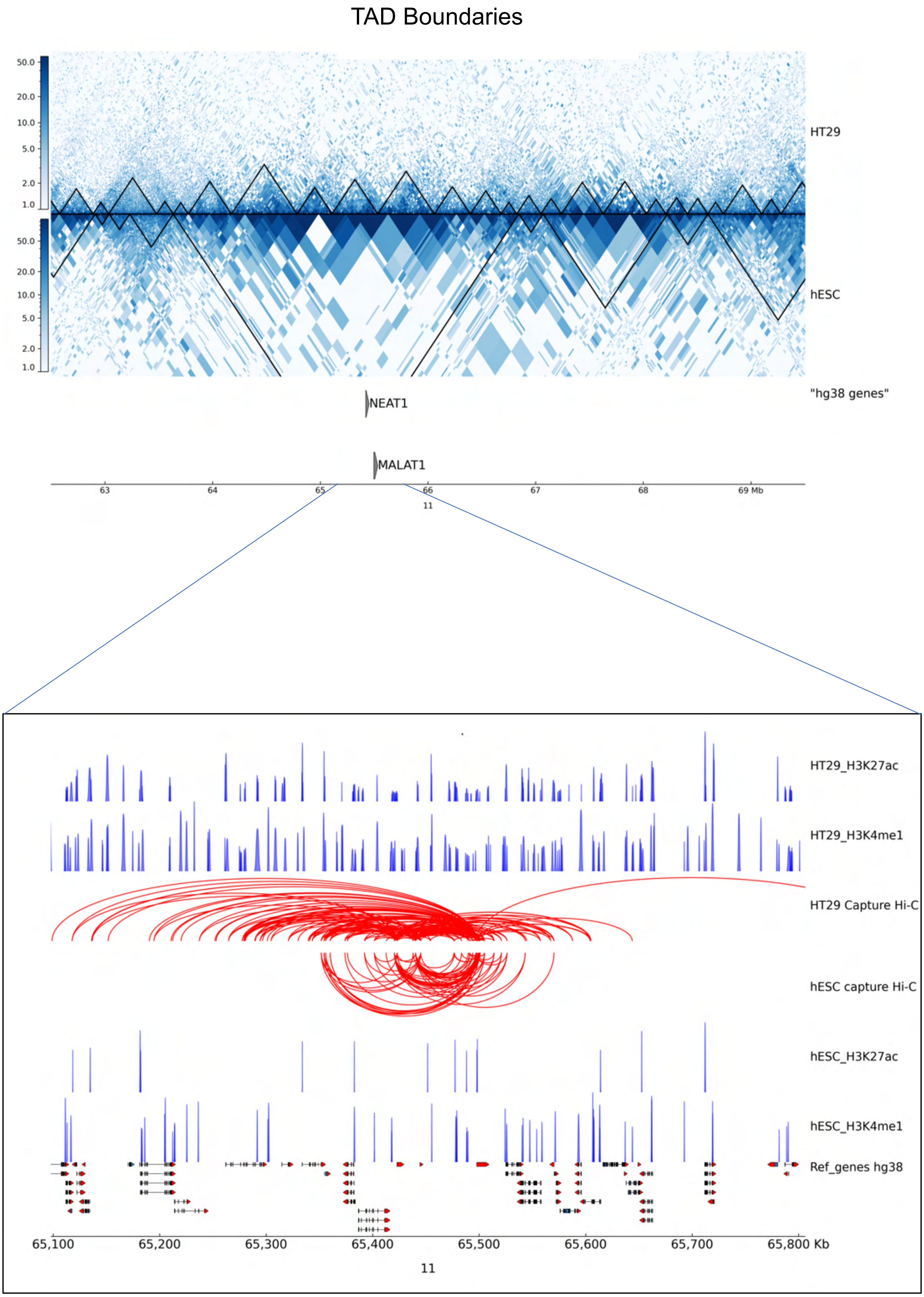
TAD Boundaries
HT29
hESC
"hg38 genes"
NEAT1
MALAT1
63
64
65
66
67
68
69 Mb
11
HT29_H3K27ac
HT29_H3K4me1
HT29 Capture Hi-C
hESC capture Hi-C
hESC_H3K27ac
hESC_H3K4me1
Ref_genes hg38
65,100
65,200
65,300
65,400
65,500
65,600
65,700
65,800 Kb
11

Figure S5 (I). Effect on gene regulation due to structural changes between cancer (HT29) versus normal (hESC) cell lines. (A) ~ 7 Mb region of chromosome 11 encompassing the MALAT1 and NEAT1 genes is shown along with TADs boundaries of Hi-C interaction maps at 10 kb resolution for case (HT29) and control (hESC). (B) Zoomed-in view of the MALAT1 and NEAT1 loci in case (HT29) and control (hESC) along with corresponding PCHi-C interaction, and ChIP-seq data for H3K27ac, H3K4me1 are displayed in blue peaks. Filtered MALAT1 and NEAT1 read counts used by CHiCAGO are displayed in red with the corresponding significant interactions shown as arcs. For clarity, only MALAT1 and NEAT1 interactions were shown.

TAD Boundaries

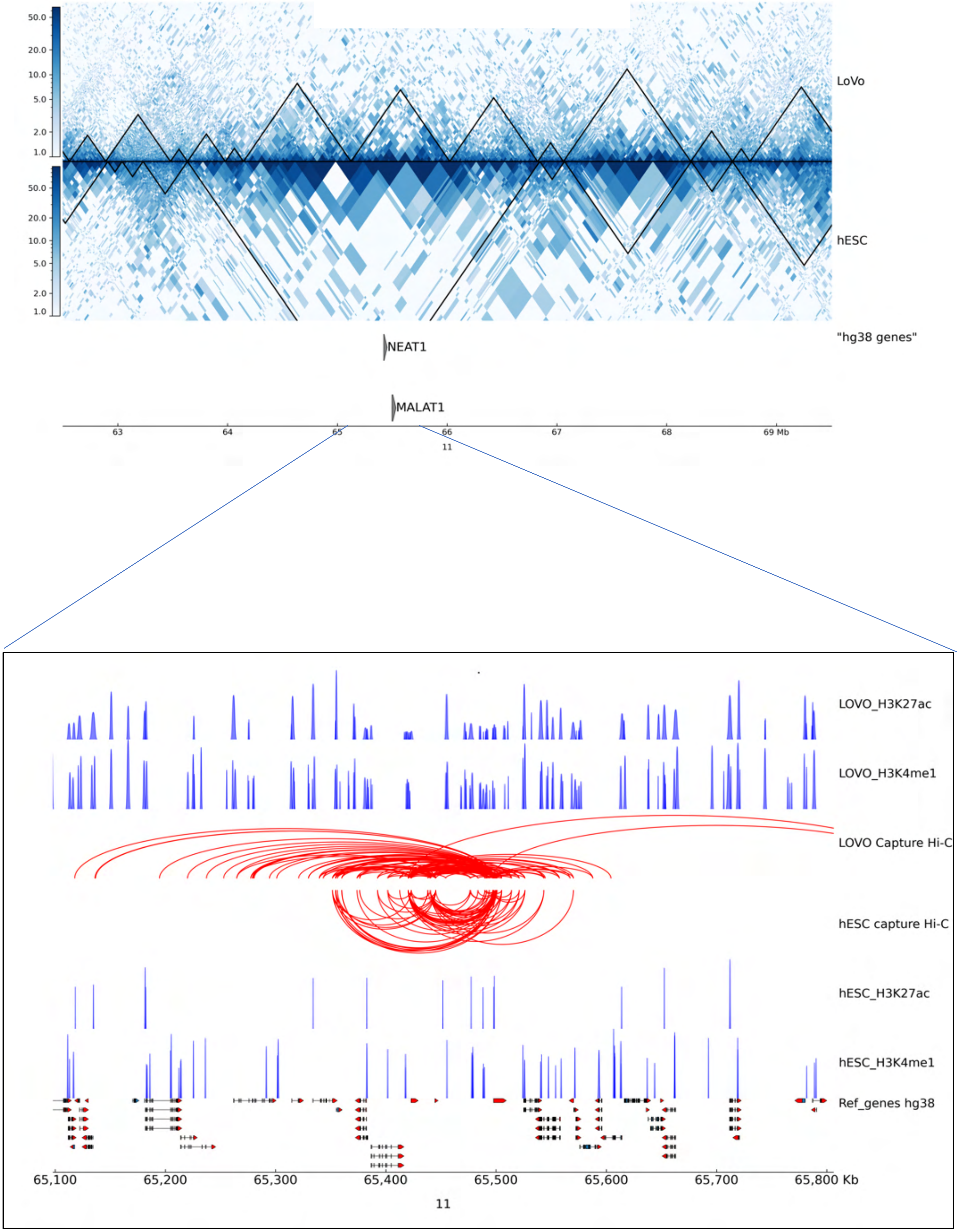

Figure S5(II). Effect on gene regulation due to structural changes between cancer (LoVo) versus normal (hESC) cell lines. (A) ~ 7 Mb region of chromosome 11 encompassing the MALAT1 and NEAT1 genes is shown along with TADs boundaries of Hi-C interaction maps at 10 kb resolution for case (LoVo) and control (hESC). (B) Zoomed-in view of the MALAT1 and NEAT1 loci in case (LoVo) and control (hESC) along with corresponding PCHi-C interaction, and ChIP-seq data for H3K27ac, H3K4me1 are displayed in blue peaks. Filtered MALAT1 and NEAT1 read counts used by CHiCAGO are displayed in red with the corresponding significant interactions shown as arcs. For clarity, only MALAT1 and NEAT1 interactions were shown.

TAD Boundaries

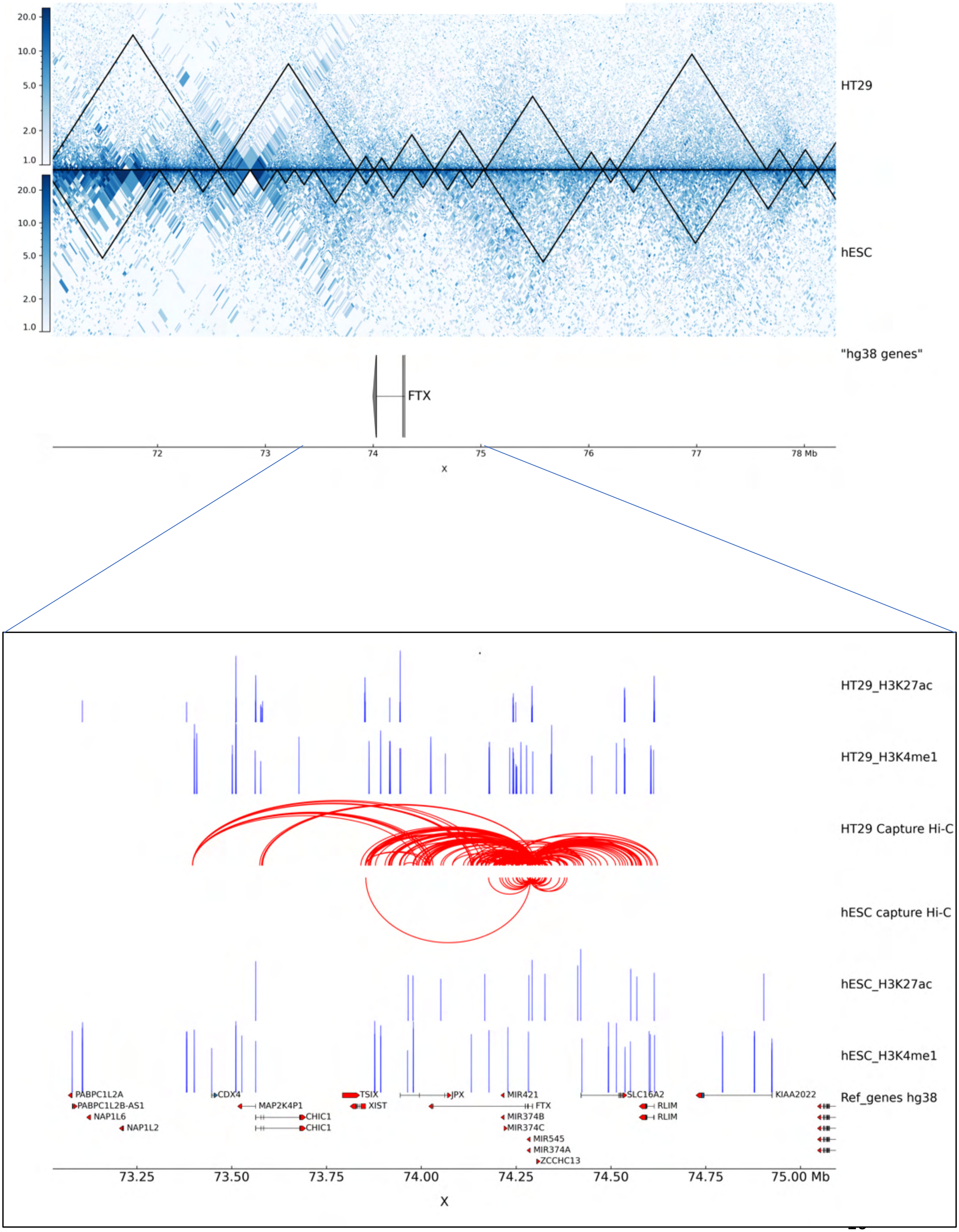

Figure S6 (I). Effect on gene regulation due to structural changes between cancer (HT29) versus normal (hESC) cell lines. (A) ~ 7 Mb region of chromosome X encompassing the FTX gene is shown along with TADs boundaries of Hi-C interaction maps at 10 kb resolution for case (HT29) and control (hESC). (B) Zoomed-in view of the FTX locus in case (HT29) and control (hESC) along with corresponding PCHi-C interaction, and ChIP-seq data for H3K27ac, H3K4me1 are displayed in blue peaks. Filtered FTX read counts used by CHiCAGO are displayed in red with the corresponding significant interactions shown as arcs. For clarity, only FTX interactions were shown.

TAD Boundaries

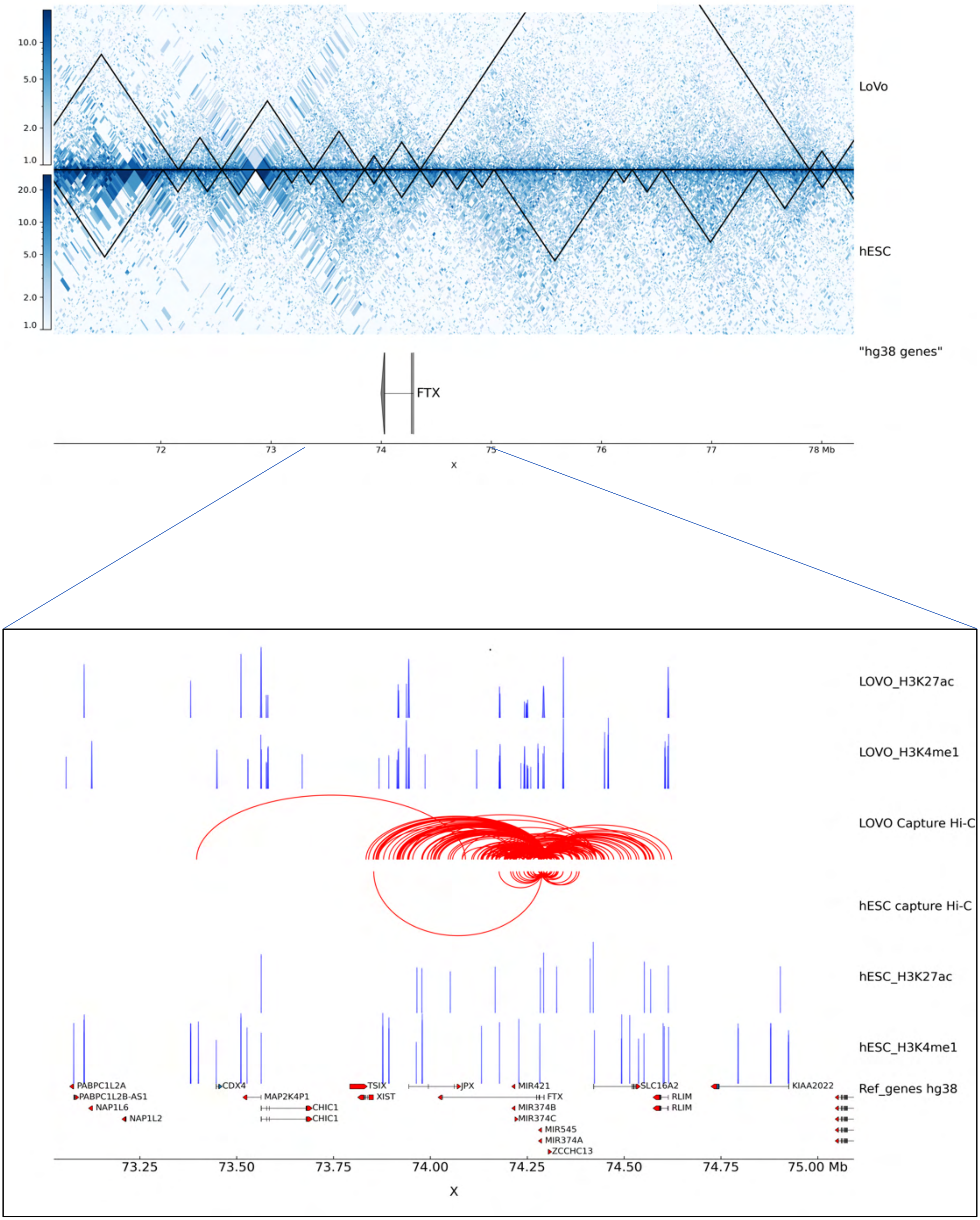

Figure S6 (II). Effect on gene regulation due to structural changes between cancer (LoVo) versus normal (hESC) cell lines. (A) ~ 7 Mb region of chromosome X encompassing the FTX gene is shown along with TADs boundaries of Hi-C interaction maps at 10 kb resolution for case (LoVo) and control (hESC). (B) Zoomed-in view of the FTX locus in case (LoVo) and control (hESC) along with corresponding PCHi-C interaction, and ChIP-seq data for H3K27ac, H3K4me1 are displayed in blue peaks. Filtered FTX read counts used by CHiCAGO are displayed in red with the corresponding significant interactions shown as arcs. For clarity, only FTX interactions were shown.

TAD Boundaries

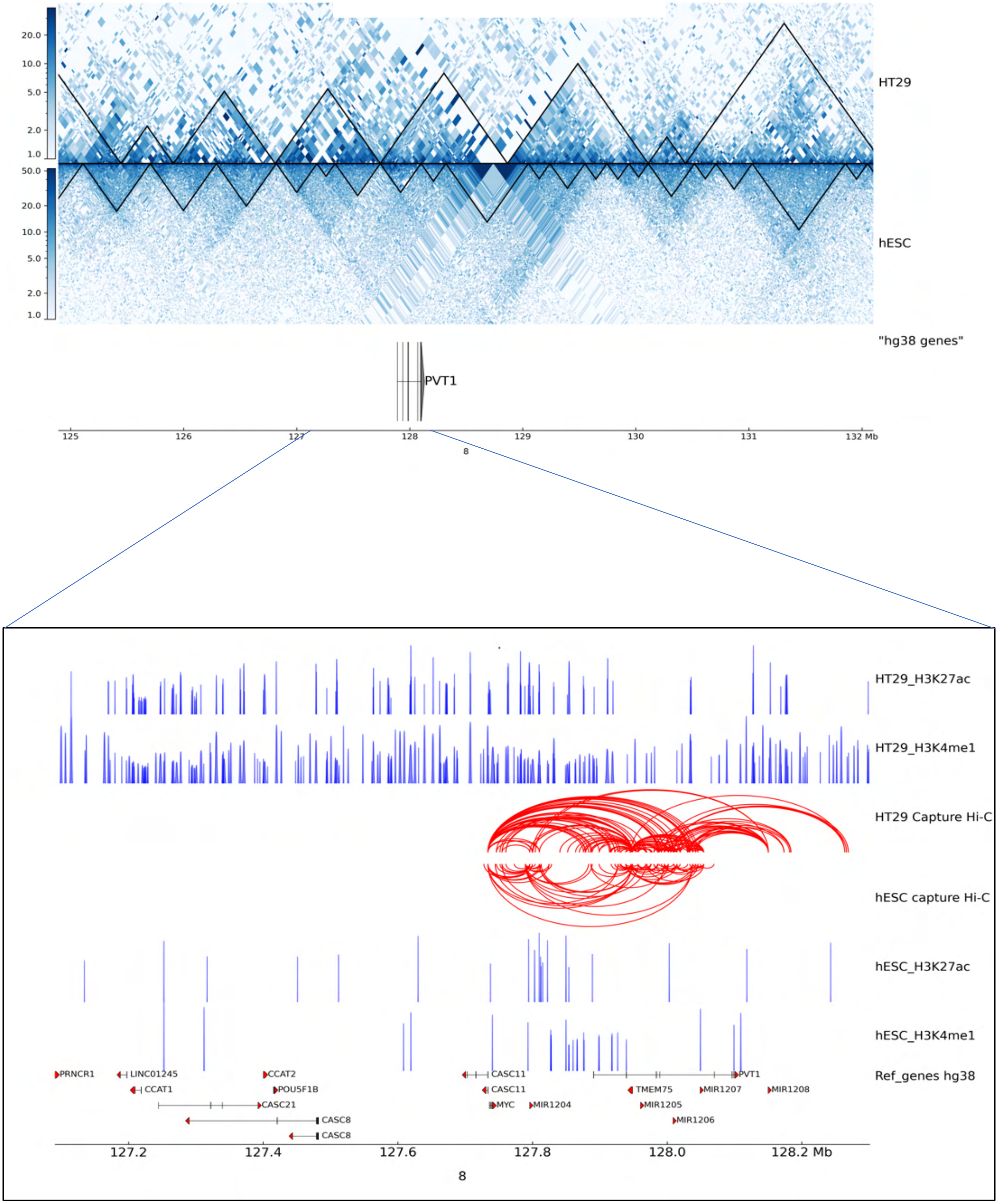

Figure S7 (I). Effect on gene regulation due to structural changes between cancer (HT29) versus normal (hESC) cell lines. (A) ~ 7 Mb region of chromosome 8 encompassing the PVT1 gene is shown along with TADs boundaries of Hi-C interaction maps at 10 kb resolution for case (HT29) and control (hESC). (B) Zoomed-in view of the PVT1 locus in case (HT29) and control (hESC) along with corresponding PCHi-C interaction, and ChIP-seq data for H3K27ac, H3K4me1 are displayed in blue peaks. Filtered PVT1 read counts used by CHiCAGO are displayed in red with the corresponding significant interactions shown as arcs. For clarity, only PVT1 interactions were shown.

TAD Boundaries

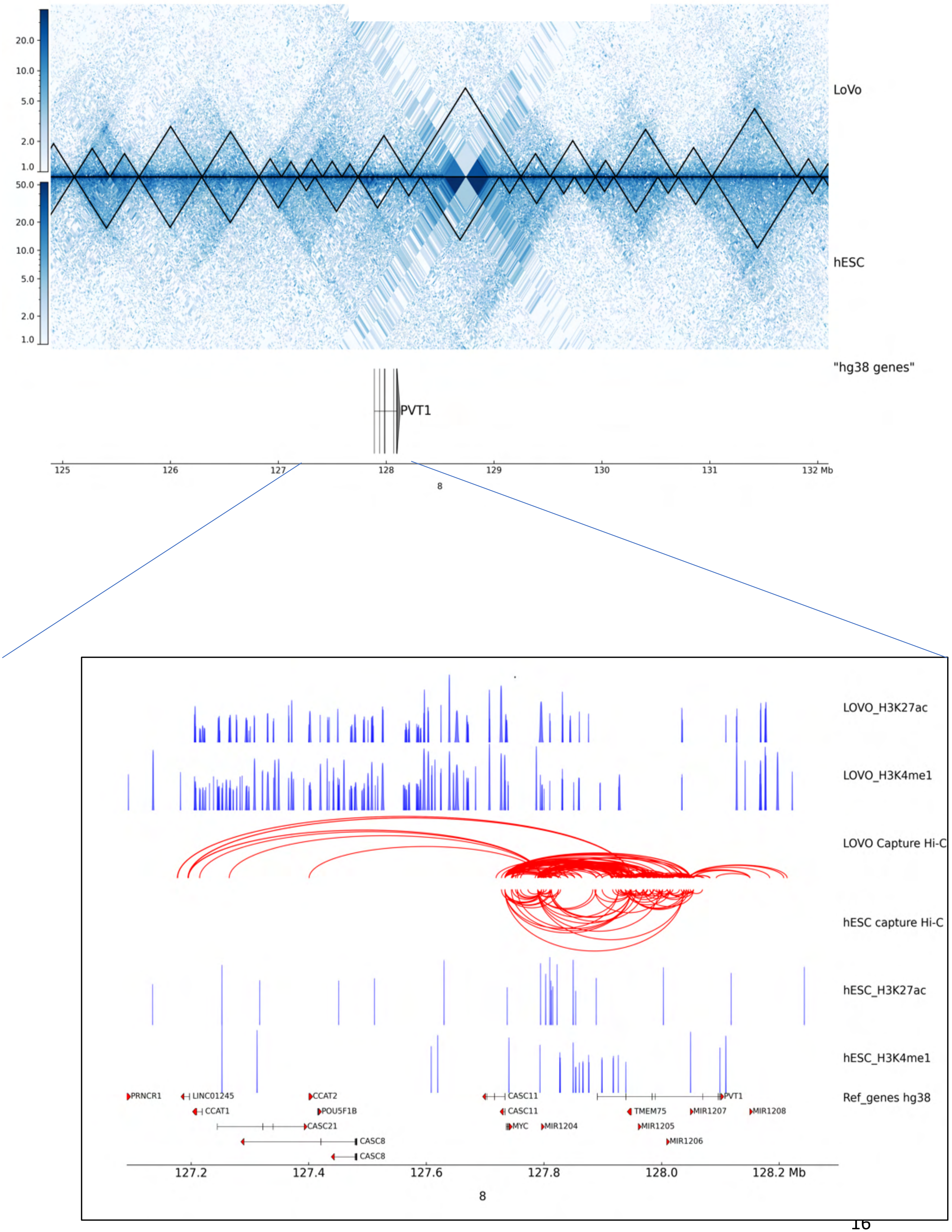

Figure S7 (II). Effect on gene regulation due to structural changes between cancer (LoVo) versus normal (hESC) cell lines. (A) ~ 7 Mb region of chromosome 8 encompassing the PVT1 gene is shown along with TADs boundaries of Hi-C interaction maps at 10 kb resolution for case (LoVo) and control (hESC). (B) Zoomed-in view of the PVT1 locus in case (LoVo) and control (hESC) along with corresponding PCHi-C interaction, and ChIP-seq data for H3K27ac, H3K4me1 are displayed in blue peaks. Filtered PVT1 read counts used by CHiCAGO are displayed in red with the corresponding significant interactions shown as arcs. For clarity, only PVT1 interactions were shown.

TAD Boundaries

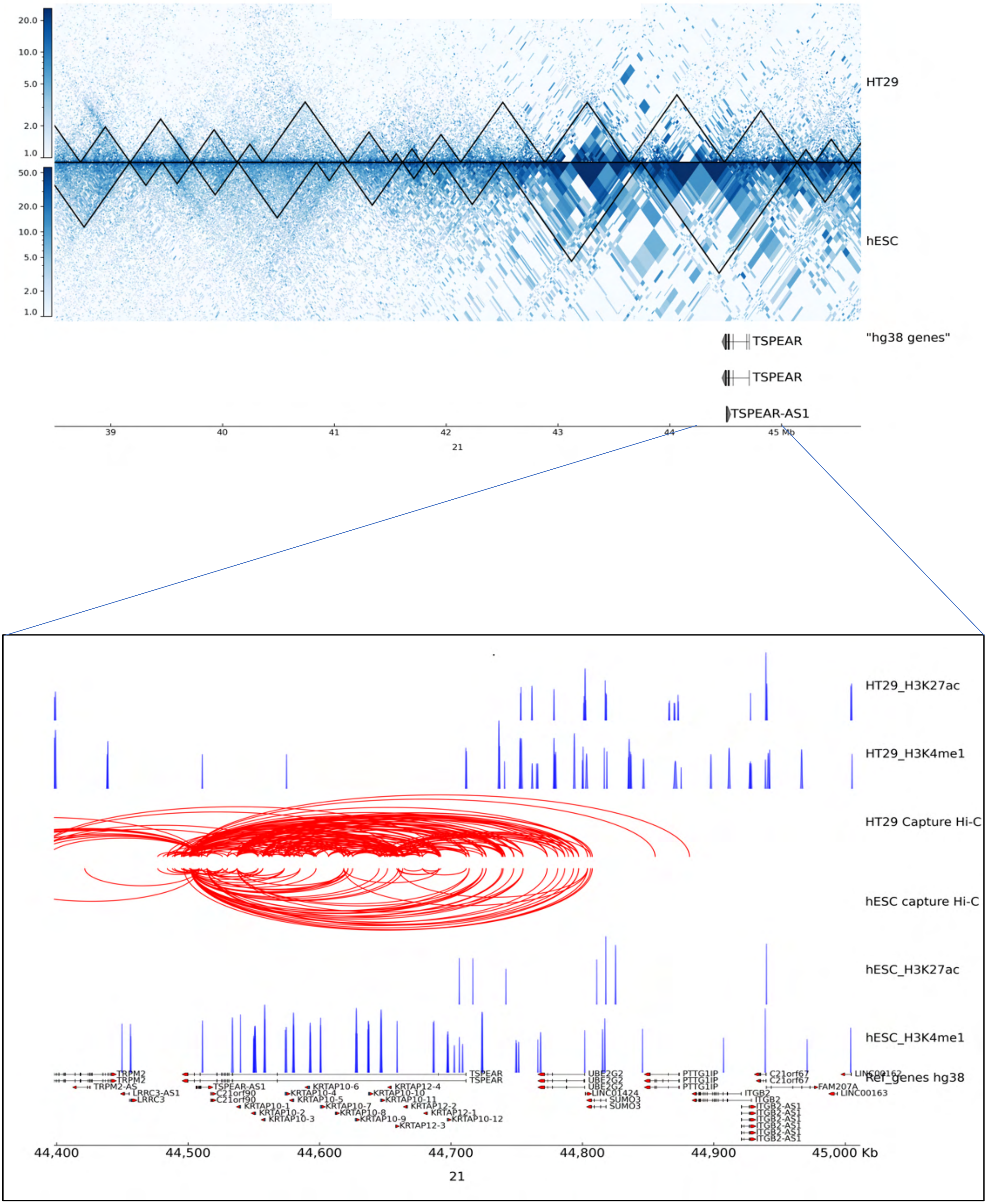

Figure S8 (I). Effect on gene regulation due to structural changes between cancer (HT29) versus normal (hESC) cell lines. (A) ~ 7 Mb region of chromosome 21 encompassing the TSPEAR gene is shown along with TADs boundaries of Hi-C interaction maps at 10 kb resolution for case (HT29) and control (hESC). (B) Zoomed-in view of the TSPEAR locus in case (HT29) and control (hESC) along with corresponding PCHi-C interaction, and ChIP-seq data for H3K27ac, H3K4me1 are displayed in blue peaks. Filtered TSPEAR read counts used by CHiCAGO are displayed in red with the corresponding significant interactions shown as arcs. For clarity, only TSPEAR interactions were shown.

TAD Boundaries

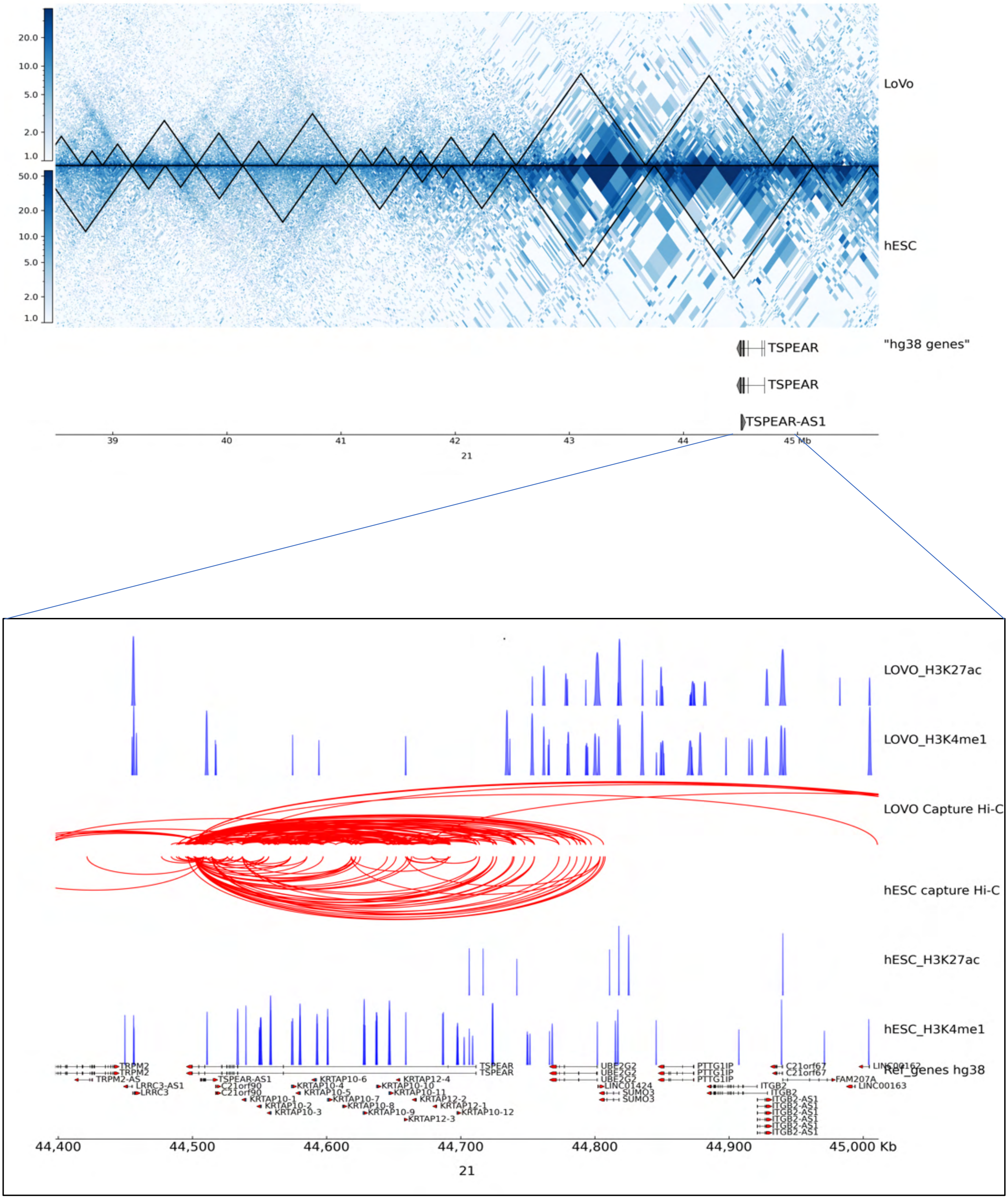

Figure S8 (II). Effect on gene regulation due to structural changes between cancer (LoVo) versus normal (hESC) cell lines. (A) ~ 7 Mb region of chromosome 21 encompassing the TSPEAR gene is shown along with TADs boundaries of Hi-C interaction maps at 10 kb resolution for case (LoVo) and control (hESC). (B) Zoomed-in view of the TSPEAR locus in case (LoVo) and control (hESC) along with corresponding PCHi-C interaction, and ChIP-seq data for H3K27ac, H3K4me1 are displayed in blue peaks. Filtered TSPEAR read counts used by CHiCAGO are displayed in red with the corresponding significant interactions shown as arcs. For clarity, only TSPEAR interactions were shown.

TAD Boundaries

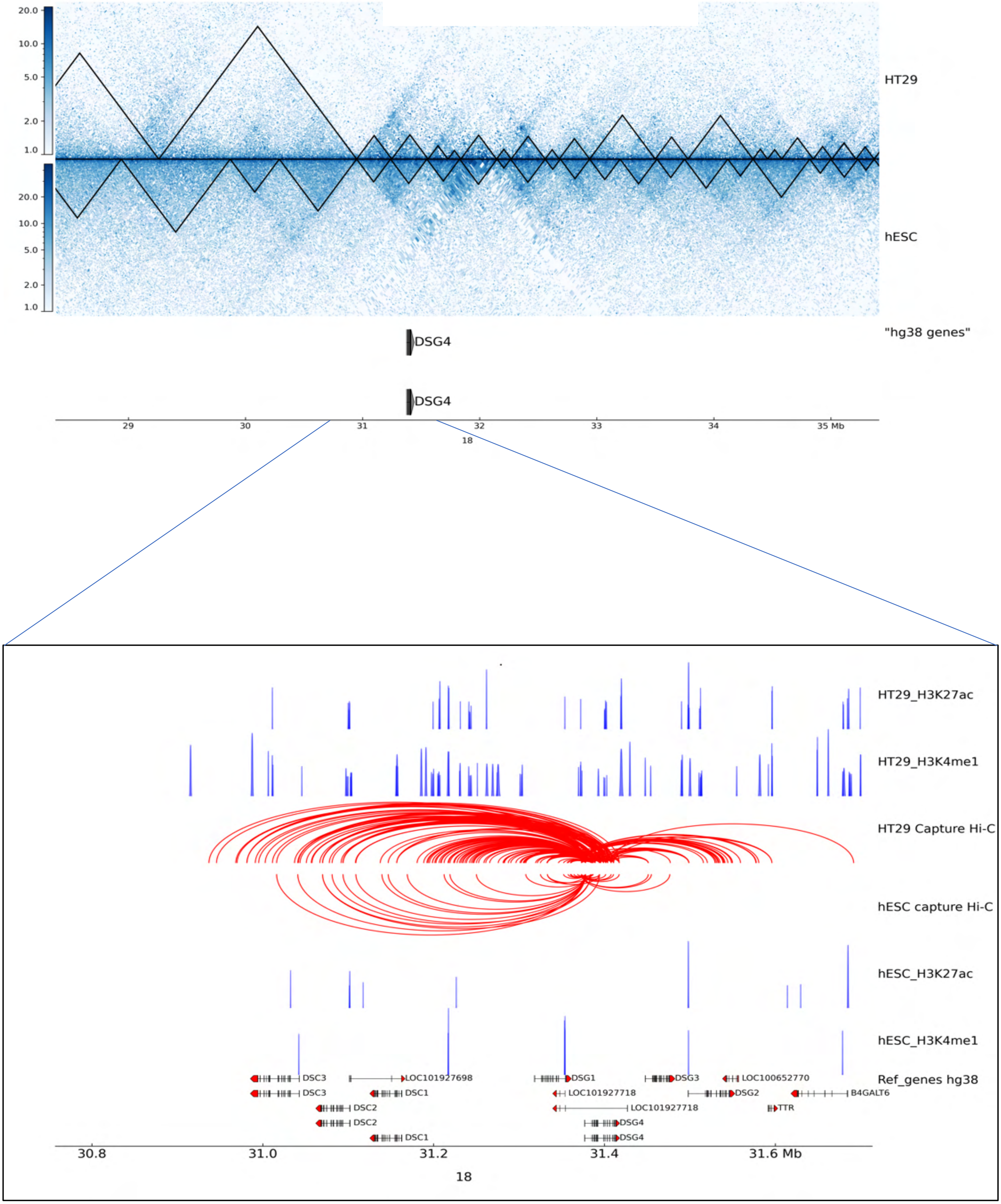

Figure S9 (I). Effect on gene regulation due to structural changes between cancer (HT29) versus normal (hESC) cell lines. (A) ~ 7 Mb region of chromosome 18 encompassing the DSG4 gene is shown along with TADs boundaries of Hi-C interaction maps at 10 kb resolution for case (HT29) and control (hESC). (B) Zoomed-in view of the DSG4 locus in case (HT29) and control (hESC) along with corresponding PCHi-C interaction, and ChIP-seq data for H3K27ac, H3K4me1 are displayed in blue peaks. Filtered DSG4 read counts used by CHiCAGO are displayed in red with the corresponding significant interactions shown as arcs. For clarity, only DSG4 interactions were shown.

TAD Boundaries

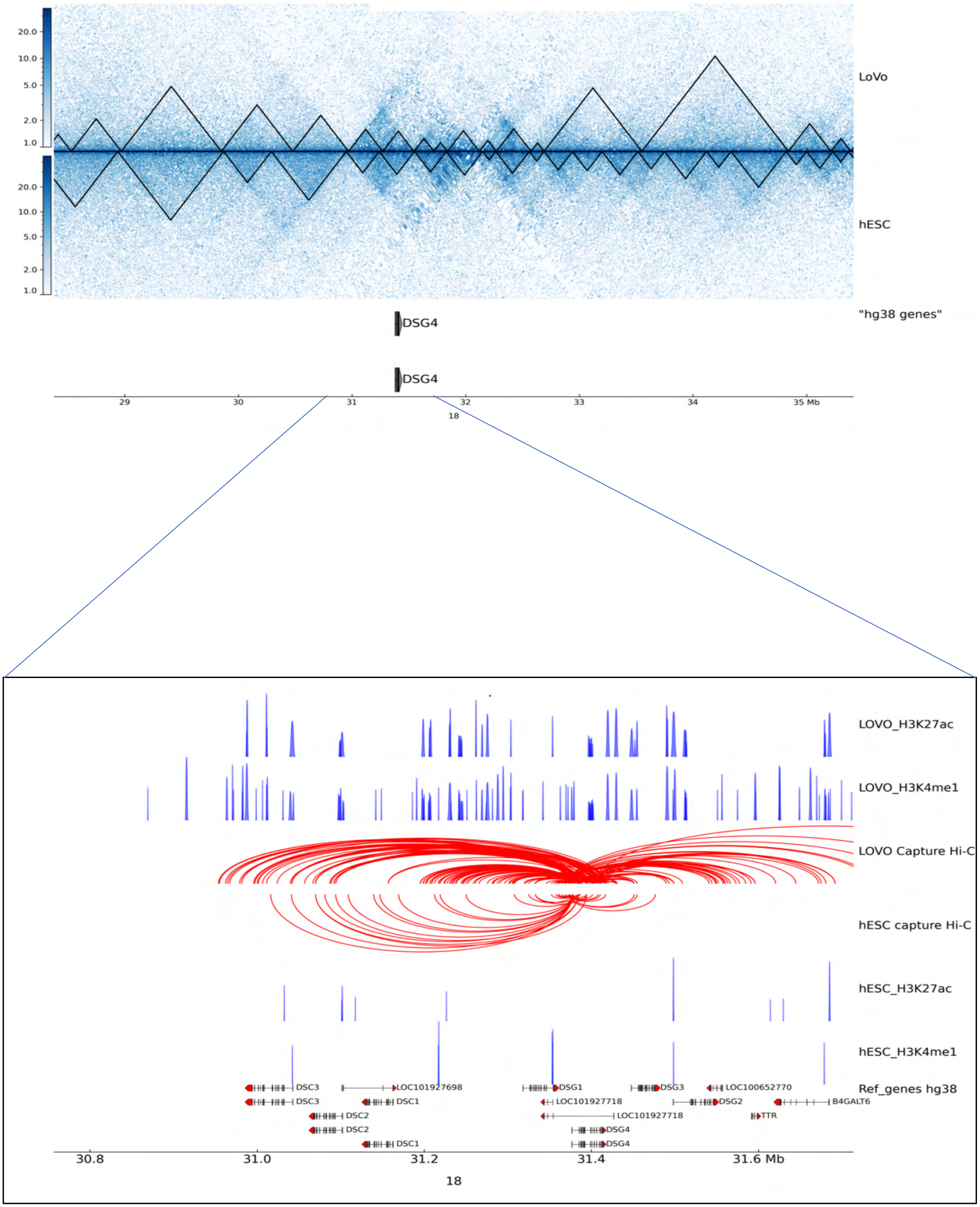

Figure S9 (II). Effect on gene regulation due to structural changes between cancer (LoVo) versus normal (hESC) cell lines. (A) ~ 7 Mb region of chromosome 18 encompassing the DSG4 gene is shown along with TADs boundaries of Hi-C interaction maps at 10 kb resolution for case (LoVo) and control (hESC). (B) Zoomed-in view of the DSG4 locus in case (LoVo) and control (hESC) along with corresponding PCHi-C interaction, and ChIP-seq data for H3K27ac, H3K4me1 are displayed in blue peaks. Filtered DSG4 read counts used by CHiCAGO are displayed in red with the corresponding significant interactions shown as arcs. For clarity, only DSG4 interactions were shown.

TAD Boundaries

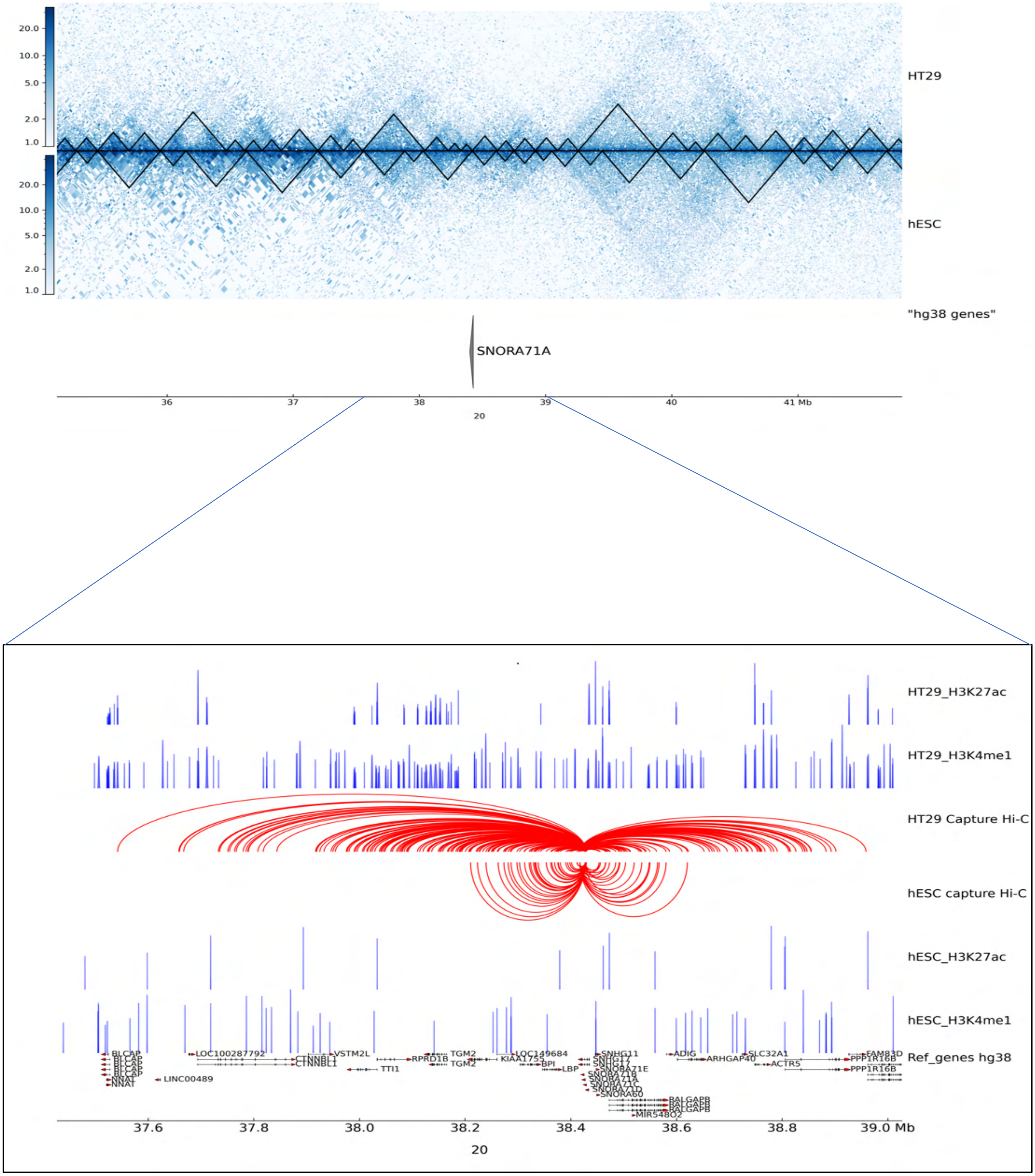

Figure S10 (I). Effect on gene regulation due to structural changes between cancer (HT29) versus normal (hESC) cell lines. (A) ~ 6 Mb region of chromosome 20 encompassing the SNORA71A gene is shown along with TADs boundaries of Hi-C interaction maps at 10 kb resolution for case (HT29) and control (hESC). (B) Zoomed-in view of the SNORA71A locus in case (HT29) and control (hESC) along with corresponding PCHi-C interaction, and ChIP-seq data for H3K27ac, H3K4me1 are displayed in blue peaks. Filtered SNORA71A read counts used by CHiCAGO are displayed in red with the corresponding significant interactions shown as arcs. For clarity, only SNORA71A interactions were shown.

TAD Boundaries

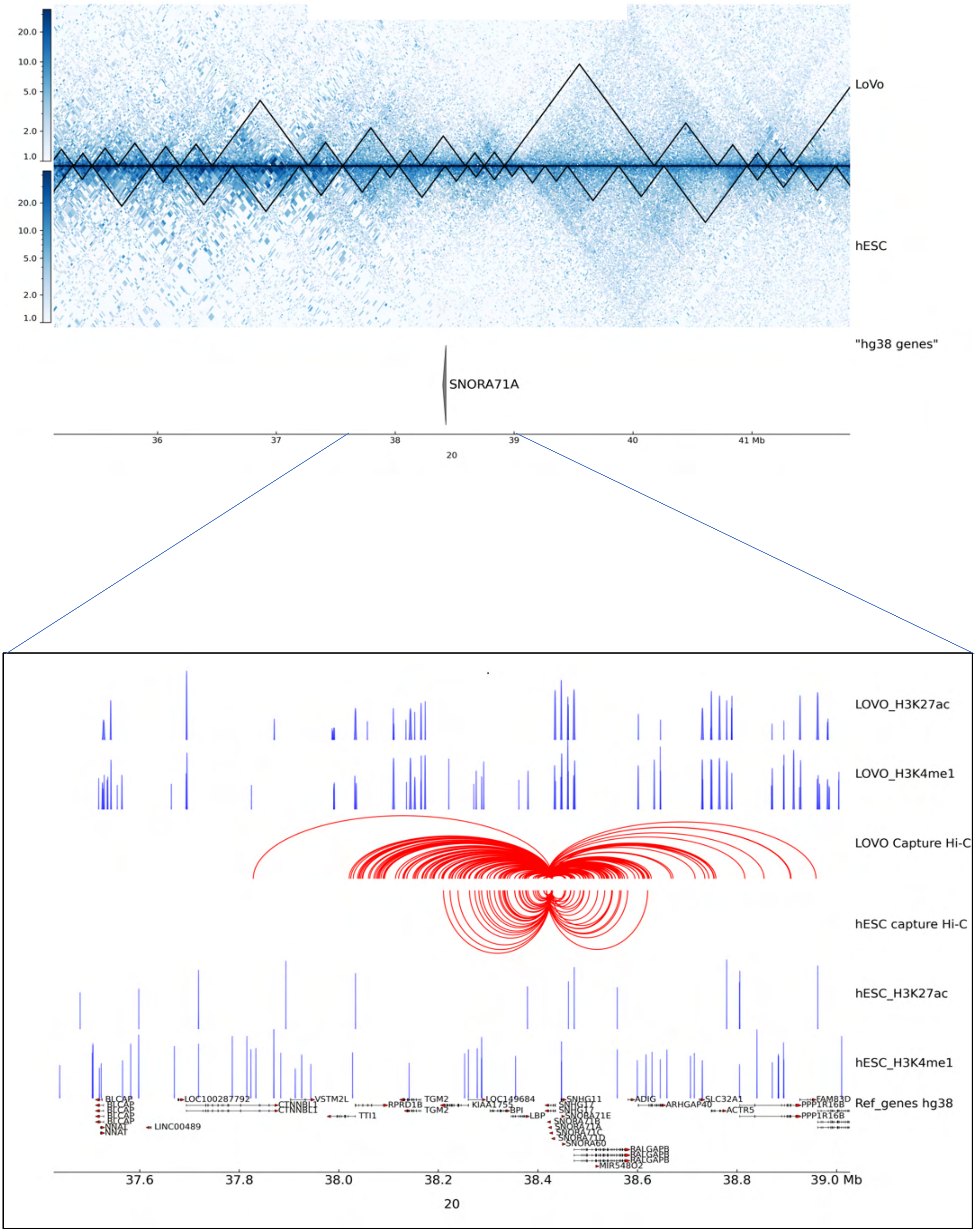

Figure S10 (II). Effect on gene regulation due to structural changes between cancer (LoVo) versus normal (hESC) cell lines. (A) ~ 6 Mb region of chromosome 20 encompassing the SNORA71A gene is shown along with TADs boundaries of Hi-C interaction maps at 10 kb resolution for case (LoVo) and control (hESC). (B) Zoomed-in view of the SNORA71A locus in case (LoVo) and control (hESC) along with corresponding PCHi-C interaction, and ChIP-seq data for H3K27ac, H3K4me1 are displayed in blue peaks. Filtered SNORA71A read counts used by CHiCAGO are displayed in red with the corresponding significant interactions shown as arcs. For clarity, only SNORA71A interactions were shown.

TAD Boundaries

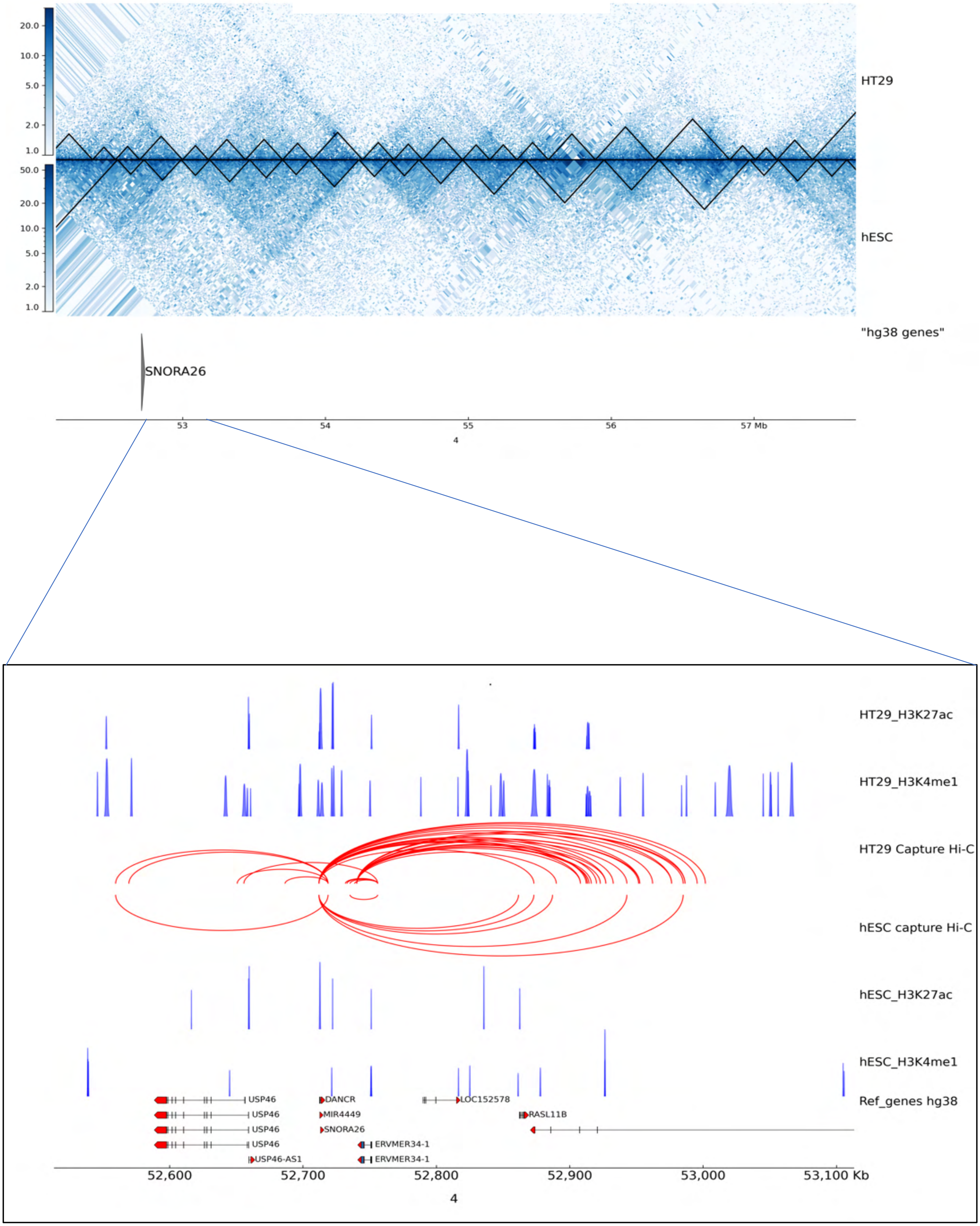

Figure S11 (I). Effect on gene regulation due to structural changes between cancer (HT29) versus normal (hESC) cell lines. (A) ~ 5 Mb region of chromosome 4 encompassing the SNORA26 gene is shown along with TADs boundaries of Hi-C interaction maps at 10 kb resolution for case (HT29) and control (hESC). (B) Zoomed-in view of the SNORA26 locus in case (HT29) and control (hESC) along with corresponding PCHi-C interaction, and ChIP-seq data for H3K27ac, H3K4me1 are displayed in blue peaks. Filtered SNORA26 read counts used by CHiCAGO are displayed in red with the corresponding significant interactions shown as arcs. For clarity, only SNORA26 interactions were shown.

TAD Boundaries

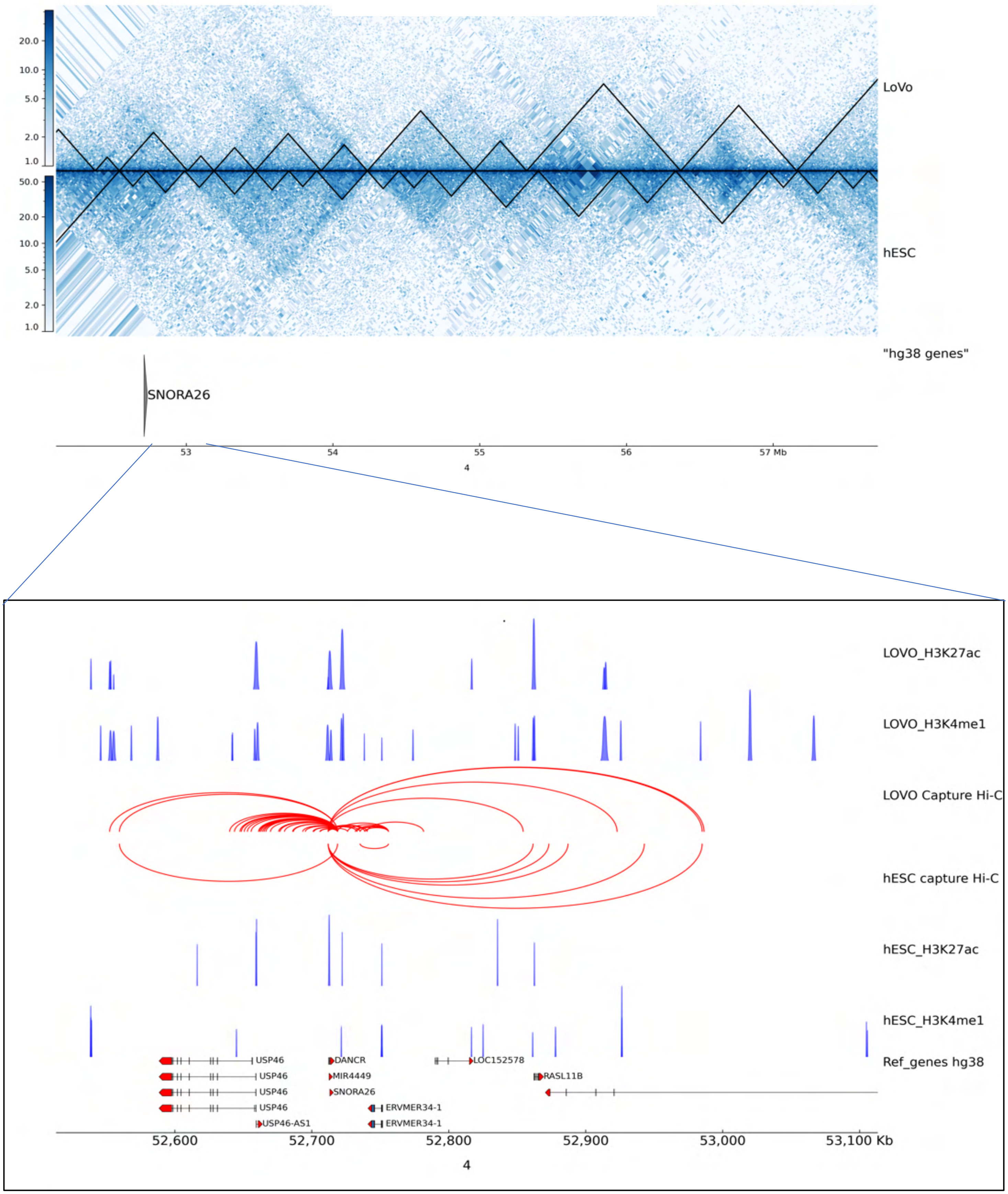

Figure S11 (II). Effect on gene regulation due to structural changes between cancer (LoVo) versus normal (hESC) cell lines. (A) ~ 5 Mb region of chromosome 4 encompassing the SNORA26 gene is shown along with TADs boundaries of Hi-C interaction maps at 10 kb resolution for case (LoVo) and control (hESC). (B) Zoomed-in view of the SNORA26 locus in case (LoVo) and control (hESC) along with corresponding PCHi-C interaction, and ChIP-seq data for H3K27ac, H3K4me1 are displayed in blue peaks. Filtered SNORA26 read counts used by CHiCAGO are displayed in red with the corresponding significant interactions shown as arcs. For clarity, only SNORA26 interactions were shown.

References:

1. Tate, J. G. *et al.* COSMIC: the Catalogue of Somatic Mutations In Cancer. *Nucleic Acids Res* **47**, D941–D947 (2019).
2. Chisanga, D. *et al.* Colorectal cancer atlas: An integrative resource for genomic and proteomic annotations from colorectal cancer cell lines and tissues. *Nucleic Acids Res* **44**, D969–D974 (2016).
3. Liu, Y., Sun, J. & Zhao, M. ONGene: A literature-based database for human oncogenes. *Journal of Genetics and Genomics* **44**, 119–121 (2017).
4. Chubb, D. *et al.* Rare disruptive mutations and their contribution to the heritable risk of colorectal cancer. *Nat Commun* **7**, 11883 (2016).
5. Rudd, M. F. *et al.* The Predicted Impact of Coding Single Nucleotide Polymorphisms Database. *Cancer Epidemiology, Biomarkers & Prevention* **14**, 2598–2604 (2005).
