# Supplementary material for: Integrated promoter-capture Hi-C and Hi-C analysis reveals fine-tuned regulation of the 3D chromatin architecture in colorectal cancer
Source: Front Genet. 2025 Mar 28;16:1553469. doi: 10.3389/fgene.2025.1553469 (PMC11985782; doi:10.3389/fgene.2025.1553469)
Supplement: Supplementary file 5 [file Table1.docx]

**Supplementary Table S1.** List of oligonucleotide primer sequences used for qRT-PCR analyses.

**Supplementary Table S2**. List of primer sequences used for ChIP-PCR analysis.

**Supplementary Table S3.** List of the transcripts along with the gene annotations. This list has been generated using TCGA colorectal cancer transcript database.

**Supplementary Table S4.** List contains enriched gene ontology and pathways for both HT29 and LoVo cell lines.

**Supplementary Table S5.** The number of overlap of bait’s interaction (region of interest) with regulatory elements such as promoter-like (PLS), proximal enhancer-like (pELS), distal enhancer-like (dELS), DNase-H3K4me3 and CTCF regions, which has been downloaded from ENCODE database (https://screen-v2.wenglab.org).
