# Supplementary material for: Integrated promoter-capture Hi-C and Hi-C analysis reveals fine-tuned regulation of the 3D chromatin architecture in colorectal cancer
Source: Front Genet. 2025 Mar 28;16:1553469. doi: 10.3389/fgene.2025.1553469 (PMC11985782; doi:10.3389/fgene.2025.1553469)
Supplement: Supplementary file 7 [file DataSheet1.pdf]

Supplementary file

(bait interaction with regulatory elements stage-wise)

**Bait interaction with↓**	**hESC/HT29**						**hESC/LOVO**					
	hESC			HT29			hESC			LOVO		
	I	II	III	I	II	III	I	II	III	I	II	III
Promoter region	20521	33115	26967	44715	65586	52929	20494	33063	21144	43437	65357	37467
Proximal enhancer region	77329	106326	83454	199317	243287	187422	76196	105711	75822	183535	225550	152083
Distal enhancer region	238403	153654	111216	926554	550096	395886	230179	146125	97088	812700	477218	318545
Dnase-H3K4me3 region	5934	4259	3177	21104	14163	10788	5718	3933	2257	18695	12831	6246
CTCF region	11391	5766	3900	48654	23452	16213	11091	5430	3620	41875	19951	13256

Table E1: Total number of bait interaction with regulatory elements stage-wise (I, II and III).

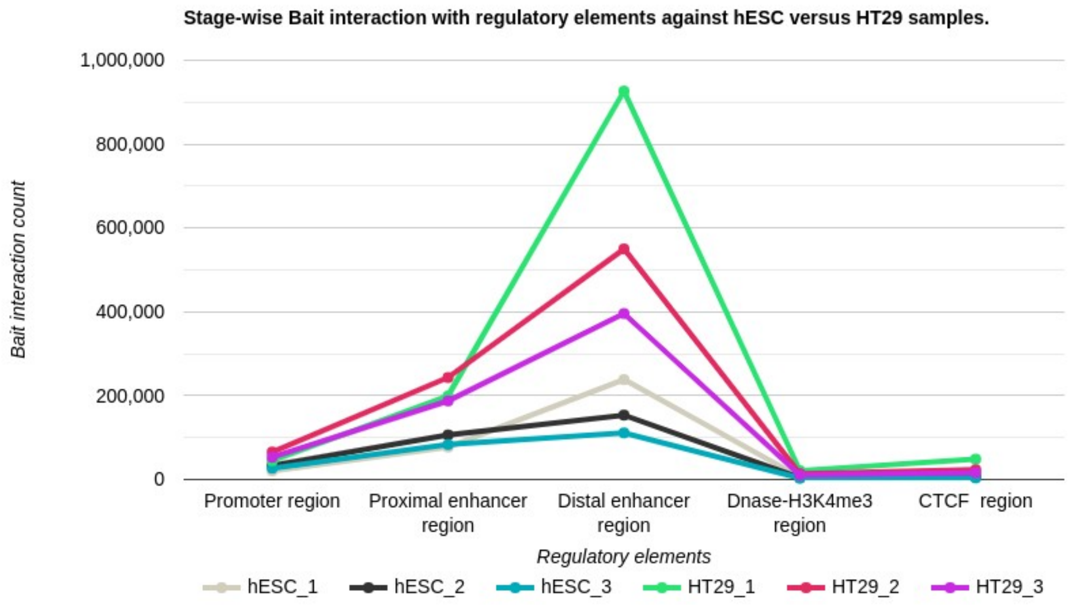

Figure E2: Stage-wise total number of bait interaction with regulatory elements (Promoter-like (PLS), Proximal enhancer-like (pELS), Distal enhancer-like (dELS), DNase-H3K4me3 and CTCF regions) hESC and HT29 cell lines in hESC/HT29.

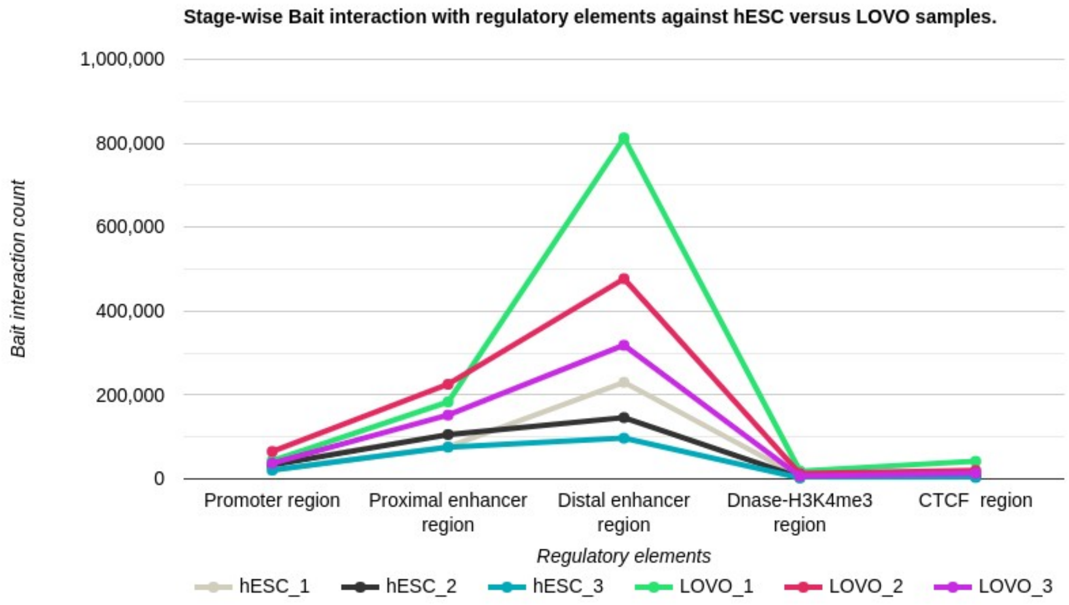

Figure E3: Stage-wise total number of bait interaction with regulatory elements (Promoter-like (PLS), Proximal enhancer-like (pELS), Distal enhancer-like (dELS), DNase-H3K4me3 and CTCF regions) hESC and LOVO cell lines in hESC/LOVO.
